# Grounding mathematics in an integrated conceptual structure, part I: experimental evidence that grounded rules support transfer that formal rules do not

**DOI:** 10.3389/fpsyg.2025.1507670

**Published:** 2025-06-03

**Authors:** Kevin W. Mickey, James L. McClelland

**Affiliations:** Department of Psychology, Stanford University, Stanford, CA, United States

**Keywords:** visuospatial representation, mathematics education, learning trigonometry, conceptual grounding, generalization, transfer, mathematical cognition

## Abstract

Mathematics relies on formal systems of rules that can be treated in isolation or grounded in a conceptual system that provides meaning for the relationships the rules express. Here, we show how the conceptual system provided by the unit circle, a visuospatial structure that provides a meaning for formal expressions in the domain of trigonometry, supports a generalizable understanding of trigonometric relationships, allowing for transfer beyond relationships explicitly taught. We examined the utility of the unit circle in our first study, in which we presented trigonometric identity problems to undergraduates (*N* = 50) who had prior coursework in pre-calculus trigonometry. Students reported using the unit circle to solve these problems more often than other approaches, and those who reported using the circle solved more problems correctly. Using other students from the same population, we then manipulated the systems they used by presenting a refresher lesson, using either formal rules or rules grounded in relationships on the unit circle (*N* = 35 in each group). Students in both conditions improved on taught problems, but only students in the grounded condition showed improvement on held-out transfer problems. Using findings from a third study further exploring the grounded condition (*N* = 64 participants), we found evidence that the circle supported transfer in two ways: by providing a procedure that could be used to solve both taught and transfer problems without rules and by allowing students to appreciate rules as capturing relationships between meaningful quantities, facilitating their application and extension. This project served as the starting place for the development of a curriculum that supports reliance on the unit circle and led to robust learning and retention of trigonometric relationships for most students with sufficient relevant prior knowledge, as described in Part II of this article.

## Introduction

A substantial body of evidence suggests that visuospatial processes may ground simple numerical and arithmetic reasoning (Dehaene, 1997; Dehaene et al., 1999). Yet higher level mathematical cognition is often treated as depending on the manipulation of structured arrangements of symbols according to structure-sensitive rules, without regard to the spatial relationships these rules may be describing (Harnad, 1990). Mathematicians and philosophers like Russell (1903), who proclaimed that “all Mathematics is Symbolic Logic”, attempted to formalize mathematics as an extension of logic, and soon after the introduction of electronic computers, symbolic rule-following programs were developed to prove logic theorems (Newell et al., 1958) and to solve mathematical equations of unbounded complexity (Martin and Fateman, 1971). Furthermore, some have argued that symbolic rule-following is essential for many types of systematic cognition (Fodor, 1975; Marcus, 2003). However, others have argued that visuospatial reasoning is a core feature of human intelligence (Wai et al., 2010), and that mathematical thinking, including achieving new mathematical insights, often relies on visuospatial reasoning (Wertheimer, 1959; Presmeg, 2006; Shepard, 2008).

Many people may agree that visuospatial reasoning and rule-based reasoning both contribute to success in mathematical problem solving. Often, however, these two forms of reasoning are considered separately and treated as drawing on distinct cognitive resources that have innate biological underpinnings. In this two-part publication, we emphasize, instead, the interdependence of formal and spatial forms of representation, the benefits of integration of these representations, and the central roles of human-invented structures and conventions in mathematical reasoning. The key points we make are the following:

- **Interdependence and integration of formal and spatial reasoning**. Formal and spatial elements are intimately interdependent in the reasoning systems employed in mathematics and mathematics education; together these elements create an integrated system in which formal and spatial representation systems work together to support problem solving and understanding.
- **Dependence of formal and spatial reasoning systems on human-invented structures and conventions**. The interdependent formal and spatial reasoning systems are human-invented systems of notation and spatial organization that allow quantities and relationships to be represented and understood in relation to each other in useful and powerful ways.
- **Benefits of integration**. Interdependence creates the opportunity to integrate strictly formal systems with more intuitive spatial reasoning systems, empowering intuition in service of mathematical understanding, promoting transfer and robust learning.
- **Challenges impeding integration**. The dependence on structured, human-invented conventions makes exploiting interdependence challenging for learners, because these systems, though powerful, rely on arbitrary, and in some ways counter-intuitive, conventions that create a barrier to understanding.

The present article—the first in a two-part series—employs experimental studies in the domain of trigonometry using undergraduates with prior exposure to the domain, yielding findings that support the idea that integration has benefits, and providing evidence that these benefits depend, at least in part, on grounding what people may often think of as formal rules in a human-invented system of spatial reasoning that provides meaning for these formal expressions. The second article in this series (Mickey et al., 2025) focuses on the challenges learners often face in mastering this system. There we describe the approach we took to addressing this challenge in an intervention study using high-school and community college students without prior exposure to the domain. We provide evidence that our approach allowed many students to learn to solve trigonometry problems that students often fail to master in typical classroom settings and to retain what they had learned after a 2–3 week delay.

In the following sections of this introduction, we ground the key points we have listed in the seminal work of Robbie Case and colleagues on the representation of whole and decimal numbers and then extend them to our domain of pre-calculus trigonometry.

### Case's concept of a central conceptual structure: an integrated representation of the mental number line

We have found it useful to consider the form of integrated representation we are describing as occurring through what Case et al. (1996) called a *central conceptual structure* which links different representations to support understanding. We argue that this kind of representation supports building the kinds of links between spatial and formal reasoning that some teachers and textbooks emphasize (Gelfand and Saul, 2001) and that many mathematicians exploit in their own patterns of reasoning (Einstein, 1979; Needham, 1998; Stewart, 1995). Because of its centrality in our work, we begin with a review of Case's views, using his example, which we hope will be accessible to many readers.

Case et al. (1996) developed and applied the idea of a central conceptual structure in his work on grade-school children's understanding of the non-negative counting numbers. Case later applied this idea to older grade-school children's understanding of decimal numbers and fractions (Moss and Case, 1999). He treated the mental number line as such a structure. For Case, the mental number line was not a simple continuum along which points can be placed, but a structured system for representing numbers, their relative positions, and the quantities they represent, all in relation to each other within a powerful set of human-invented conventions.

Though an intuitive sense of a continuum of values varying in magnitude may have some evolutionary basis (Dehaene, 1997), the mapping of such a continuum onto space has come to be construed by many investigators as an invented human representational scheme that is interdependent with a person's representation of symbolic number (Núñez, 2011; Link et al., 2014; Kanayet et al., 2018). Learning this mapping, Case argued, is an important step in the emergence of basic mathematical reasoning abilities. The number line from 0 to 10 provides the conceptual core of this system, aligning the arbitrary names and graphic symbols for the integers from 0 to 10 according to a sequence laid out in space by assigning each successive digit to each successive position in a conventionalized order from left to right, so that their relative positions with respect to each other can be considered, and allowing the quantities they stand for to be visualized by, for example, raising a finger or adding a token to a set for each increment of position along the line.

Further, Case understood the number line to have higher-order structure dependent on a very important human notational invention: The place-value system, specifically the base 10 system underlying arithmetic as it is taught around the world today. The place-value system allows numbers to be seen as sums of integer multiples of powers of 10. This system was an important human invention that makes arithmetic operations highly systematic, so that completely formal procedures can be used to perform exact numeric calculations. The number line from 0 to 100 becomes, in this framework, a structure consisting of a nesting of 10 instances of the number line from 0 to 10 within itself, and this system can be extended to larger and larger powers of 10, to represent indefinitely large quantities. There is an important arbitrary convention associated with this, in that the right-most digit in the representation of an integer quantity always represents the quantity of individual units, with each successive digit to the left representing the number of multiples by the next larger power of 10, so that, for example 173 = (1 × 100) + (7 × 10)+3.

A further extension of this human invention allows unit quantities to be partitioned to represent quantities between the unit values, now using the integers from 0 to 10 to partition the interval between 0 and 1 into ten parts. The process can be applied recursively to create finer and finer partitions. Importantly, this partitioning must be aligned with a human-invented representational convention for decimal numbers, wherein each successive digit to the right of a decimal point corresponds to the next recursive subdivision of the interval specified by its predecessor to its left. The first digit after the decimal point places the number within a specific tenth of the whole line, while the second places it within a specific tenth of that tenth of the line, and so on. In this way quantities of any magnitude and any degree of precision can be mapped into a common frame of reference and positioned with respect to each other, facilitating reasoning and problem solving about number.

In their work within this framework, Case and colleagues promoted the idea that children gradually acquire an integrated understanding of number notation, position along a structured linear dimension, and the quantity for which each number stands, as well as an understanding of relationships among various numbers, such as the understanding that 7 is between 6 and 8; that 8 references a larger quantity and a position closer to 10 than 7; that 37 can be understood as 3 tens and 7 ones and at the same time as a position along a line from 1 to 100 between 30 and 40, 3 unit steps to the left of 40. Moss and Case (1999) extended this framework to create a method for teaching decimals and fractions, starting with the number line from 0 to 100 and using it to represent percentages of a unit quantity. Here again, coordinating arbitrary conventions is key. For decimals, prior to understanding this system, children often initially treat 0.173 as a larger number than 0.37, but after being taught the relevant conventions, they realize that the former is between 0.1 and 0.2 while the latter is between 0.3 and 0.4, making the former the smaller rather than the larger number. A key part of this work was an emphasis on relating fractions to the number line, starting with 1/2 corresponding to 0.5 or 50 percent and 1/4 corresponding to 0.25 or 25 percent. Interestingly it is found in studies of children's marking of lines from 1 to 100 that 0.5, and at later ages, 0.25 and sometimes 0.75, are treated as landmarks or reference points around which other points are placed within this system (Barth and Paladino, 2011; Sullivan et al., 2011).

Ideas building on Case's insights have had an important impact on early mathematics eduction. A seminal body of work starting with Ramani and Siegler (2008) drew on Case's ideas to show that a game in which children compete to move a token from a start square to a finish square across a row of squares labeled with the digits from 1 to 10, while tracking each successive square's name as the next digit in the series, leads to marked improvement in several early number skills including the ability to judge which of two numbers is larger. A considerable body of later work reviewed in Siegler and Lortie-Forgues (2014) draws on and extends these ideas to numbers of larger magnitudes, as well as fractions and negative numbers.

### The unit circle: an integrated conceptual structure in trigonometry

Drawing on Case's ideas, we investigate the grounding of mathematical understanding in what we believe Case would have called a central conceptual structure in the domain of pre-calculus trigonometry, a branch of mathematics often taught in pre-calculus courses at the gateway to advanced mathematics. This subject matter sits at the intersection of algebra and geometry and offers rich opportunities for investigating visuospatial and rule-based reasoning in productive mathematical thinking. The concepts introduced in this material are foundational to many fields of science and engineering, going far beyond the basic “triangle trig” relationships often taught in conjunction with a first course in geometry.

Pre-calculus trigonometry is also a domain that can be very challenging for many high-school students, as we document in the sequel to this article (Mickey et al., 2025), and so presents a real opportunity for educational advances. Finding better ways of teaching trigonometry may be an important way to remove some of the barriers to STEM careers, potentially leading to insights that could support better student learning in many branches of mathematics, science, and engineering.

In pre-calculus trigonometry, students learn about the trigonometric functions, including the sine (sin), cosine (cos), and tangent (tan) functions, using them to solve problems such as determining the distance between two spatially remote objects or the value of an oscillating quantity such as the height of the sun in the sky. These functions can be conceptualized in many different ways: They can refer to ratios of the lengths of pairs of sides of right triangles or to the values of functions that oscillate regularly as a function of time. They also enter into many complex relationships with each other–relationships that are specifically taught as “identities” and that are central to reasoning in trigonometry.

Identities are relationships that hold regardless of the specific value of the argument to a function. Two of the most basic identities are:


$$\begin{array}{l} \sin\left(-\theta \right)=-\sin\left(\theta \right) \end{array}$$

and


$$\begin{array}{l} \cos\left(-\theta \right)=\cos\left(\theta \right), \end{array}$$

where θ can be any real number. These identities can seem arbitrary. In one, the minus sign inside the parentheses appears to have been pulled out and placed in front of the function definition, while in the second, the minus sign has simply disappeared. Why this difference, and why is it in cos and not sin that the minus sign disappears? Indeed, as we will document, undergraduates at Stanford University who have taken high school classes covering pre-calculus trigonometry often make mistakes answering simple multiple choice questions about the second of identities, choosing −cosθ rather than cos(θ) to be equal to cos(−θ). However, these relationships are not at all arbitrary–they have meaning when understood within the context of a conceptual framework known as the unit circle, which we now describe.

**Figure 1:**
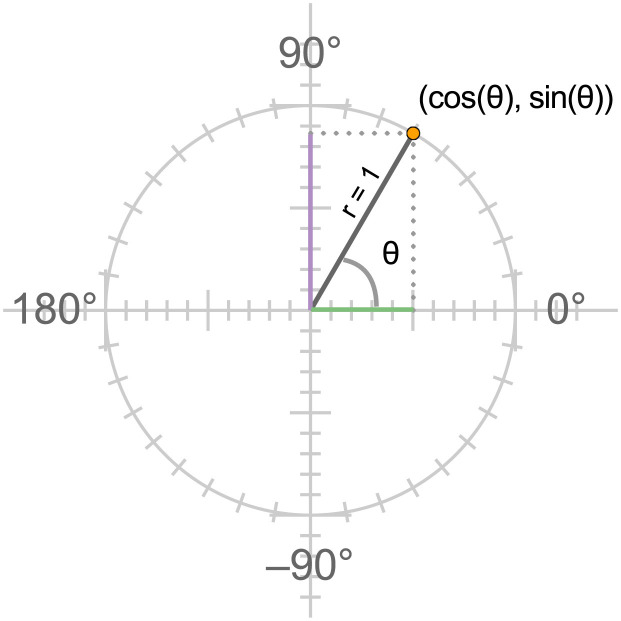
The unit circle. In triangles, cosine is defined as the ratio of the length of the side adjacent to the angle (shown in green in the figure) to the length of the hypotenuse, and the radius of the unit circle is equal to 1. The ratio then reduces to the length of the horizontal side. Similarly the sine ratio reduces to the length of the vertical side (shown in lavender). On the unit circle, these quantities correspond to the values of the x and y coordinates of the point where the terminal side of the angle intersects the circle.

The unit circle, shown in Figure 1, has a radius of 1, and its center is at (0, 0) on the (x, y) coordinate plane. Angles can be visualized on the unit circle by defining one side (called the initial side) as the positive side of the x-axis, and by rotating the other (terminal) side of the angle (a ray with one end fixed at the center of the circle) up (counter-clockwise) for positive angles or down (clockwise) for negative angles. To measure angles one needs a unit of measurement. When these relationships are first introduced the unit of measurement is one degree, a unit that divides the circle into 360 equal parts. In this system, the cosine of an angle corresponds to the x-coordinate of the point where the angle's terminal side intersects the circle, and the sine of an angle is equal to this point's y-coordinate.

These unit circle definitions of sine and cosine extend the definitions these functions have in right triangles, where the absolute values of the x and y coordinates of the endpoints of the terminal side of the angle correspond to the lengths of sides of a right triangle, as illustrated in Figure 1.

**Figure 2:**
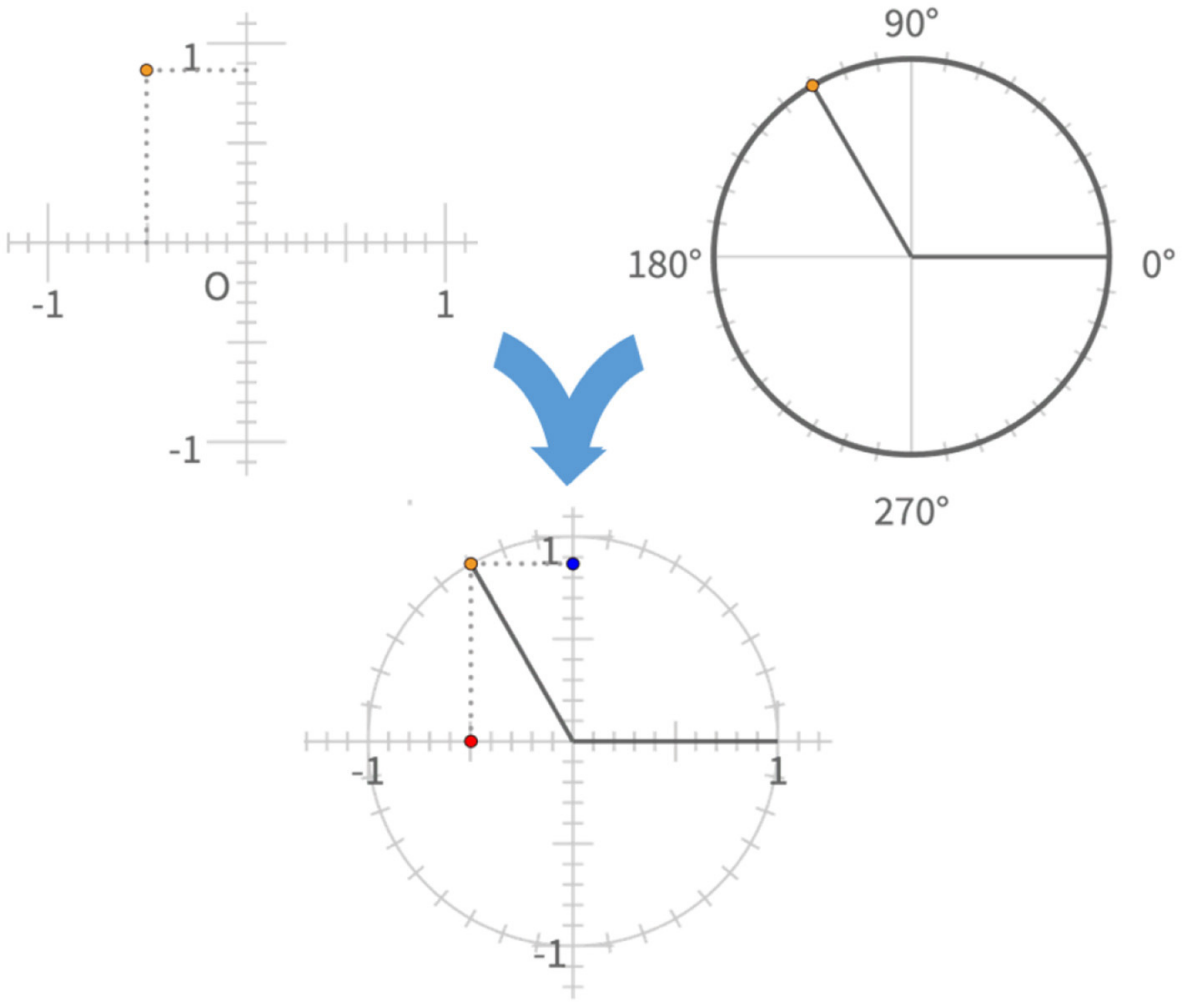
The unit circle is a conceptual structure in trigonometry that integrates the system for representing positions of points on the X-Y coordinate plane with a system for representing positions around the circumference of a circle.

We propose that the unit circle serves as an integrated conceptual structure in trigonometry, combining the conceptual system for representing the horizontal and vertical positions of points on the plane in terms of real-valued [x,y] coordinate pairs with the conceptual system for representing points on a circle in terms of a single real-valued angular coordinate θ (Figure 2). We use the word *integrated* rather than Case's term *central* to emphasize how it brings two distinct conceptual systems into coordination with each other. Furthermore, each of these conceptual structures can be thought of as an integrated structure that combines geometric and numerical content (Fauconnier and Turner, 1998), with the unit circle integrating both of these into a higher-order conceptual blend (Lakoff and Nunez, 2000).

Fauconnier and Turner (1998) argued that productive insights can emerge from conceptual integration by bringing elements of distinct structures into direct relationship with each other (e.g. the position of a point on the circle relative to reference positions on the circle and on the plane) and combining these with even more basic knowledge brought into the representation by a process they call “completion”. A key point to note in this context is that, just as with Case's conception of the number line, each of these systems has arbitrary conventions that must be mastered. The *x* and *y* coordinates of the Cartesian plane each consist of the extended number line we discussed above, the one called the *x* axis running from left to right in ascending numerical value with 0 at the center of the circle, and the other called *y* running from bottom to top with 0 at its intersection with the other. The placement of the horizontal coordinate first in an [*x, y*] coordinate pair is an arbitrary convention. The θ coordinate on the circle has an arbitrary, conventionalized reference point where the circle intersects the positive end of the *x* axis of the Cartesian plane, its positive direction is arbitrarily counter-clockwise rather than clockwise, and it divides the circle arbitrarily into 360 parts. Its most important reference points are now located at 90, 180, and 270 degrees, corresponding to the three points other than 0 where the circle intersects an axis of the Cartesian plane.

**Figure 3:**
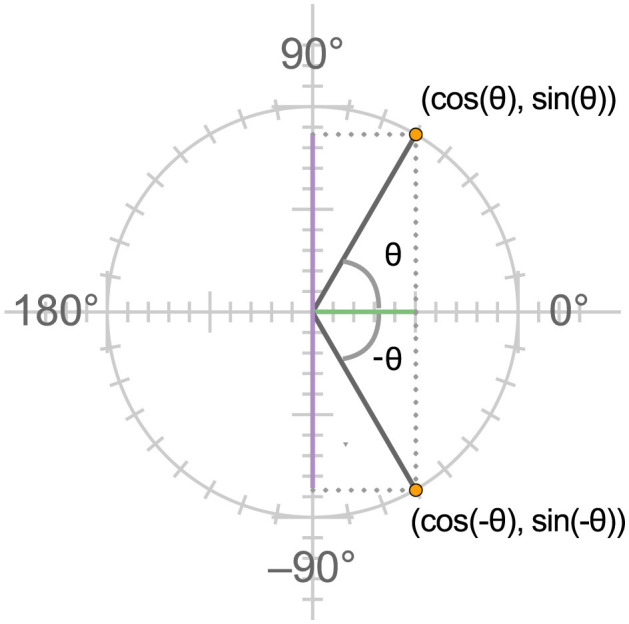
Using the unit circle to understand why sin(θ) = −sin(θ) while sin(θ) = −sin(θ). The first expression can be read as stating that the position reached by rotating a point in the negative direction from the reference point at 0 degrees on the positive *x* axis is the same distance from the *x* axis as the point reached by an equal rotation in the positive direction, but in the opposite direction, while horizontal distance of these points from the *y* axis are the same.

If a person understands both the Cartesian and circular coordinate systems, and the way in which they are coordinated in the unit circle, the identities sin(−θ) = −sin(θ) and cos(−θ) = cos(θ) become meaningful, as visualized in Figure 3. The first corresponds to the statement that a rotation of θ degrees in the negative direction from the reference point on the horizontal *x* axis through the circle will place a point at the same vertical distance from the reference point but in the opposite direction from a rotation of the same number of degrees in the positive direction. The second corresponds to the statement that the *x* coordinates of the points reached by these equal and opposite rotations will be the same. These correspondences can be visualized on the unit circle. Another identity, such as the identity cos(θ+180) = −cos(θ), can be understood as expressing the idea that the horizontal position of the point reached by rotating half way round the circle from any position θ will be the same horizontal distance from the center of the circle as the point at θ but in the opposite direction. Visualizing the relevant positions on the circle and considering their horizontal and vertical positions grounds the relationships and makes them visually apparent. The evidence we will present below suggests that this supports understanding these identities, in a way that promotes transfer to other related identities.

### Overview

In the present article, we present experimental studies investigating the role of visuospatial representations, formal rules, and their interaction in solving—and learning to solve—trigonometric identity problems. Our findings suggest that grounding in the unit circle supports robust, generalizable understanding, and that it does so, at least in part, by allowing the use of grounded rules—rules that describe meaningful spatial relationships—that generalize because the relationships involved are more general than those captured in specific taught examples. Our findings also suggest that success in using this model is far from universal, even among the undergraduates at Stanford, a highly selective university, all of whom had prior exposure to the unit circle in pre-calculus trigonometry. Specifically, only a subset of students relied on the unit circle spontaneously, and only a subset of those who did not do so benefited from a reminder lesson grounding trigonometric identities in the unit circle. In the *General Discussion*, we return to this theme.

In a related project we began after completing the second study described in the present article, we found that success was even more limited among students in a pre-calculus class at a public high school near the university. In the second article in this series (Mickey et al., 2025), we consider the reasons for this, and we build on what we learned in the studies in the present article and on principles and insights from others' research on the psychology of learning and education to develop a curriculum that supported robust learning of trigonometric relationships in a substantial proportion of students without prior exposure to this material.

## Study 1: exploring trigonometric reasoning

To explore what representations people use to solve trigonometry problems, we began with an preliminary study to develop testable hypotheses, using 37 undergraduate students taking an introductory psychology class at Stanford University. Participants first solved a set of 40 identity problems described more fully in the methods for Study 1 below, then answered a few short questions and took a short break, then completed forty more such problems before answering a fuller set of questions about the representations they used and their trigonometry background.

In this study, we found a wide range of variation in performance, with many students scoring below 50% correct overall, even though all of the participants had some prior exposure to trigonometry and strong enough quantitative backgrounds to gain admission to a highly selective university. Strikingly, participants performed especially poorly on a problem that taps a basic and explicitly instructed aspect of pre-calculus trigonometry, namely the value of the cosine of a negative argument. Given a probe expression like cos(−70+0), where the numbers represent the arguments to the cosine function in degrees, and the task of identifying which of the alternatives sin(70), −sin(70), cos(70), and −cos(70) is equivalent to the probe expression, many participants chose −cos(70), whereas the correct answer is cos(70).

Our preliminary study also provided evidence suggesting a role for visuospatial representations in trigonometry. Specifically, we found that students reported using one of the visuospatial representations we asked them about—the unit circle—more frequently than others, and those who reported using the unit circle tended to perform better at solving the identity problems. Participants reported using the unit circle more than any other representation; self-reported circle use was more strongly associated with greater overall accuracy than other representations, even after controlling for self-reported number of courses involving trigonometry and years since last use of trigonometry; and those who reported always using the unit circle did much better on cos(−θ+0) problems than other participants.

Our preliminary findings suggest a central role for the unit circle in solving the trigonometric identity problems used in our study and point to a specific role for this representation in facilitating mastery of fundamental aspects of trigonometry. We therefore set out to establish the reliability of the association between unit circle use and performance on trigonometric identity questions and to determine whether a brief lesson grounded in the unit circle could produce benefits relative to a lesson relying on formal rules. Our first two studies establish that unit circle use is indeed associated with better performance on trigonometric identities questions and demonstrate that a brief lesson grounded in the unit circle leads to performance gains that transfer to untaught problems, whereas a rule-based lesson does not. Our third study then builds on these results to explore possible explanations for the transfer benefit of the unit-circle based lesson.

Study 1 explored the relationship between reported circle use and performance on our trigonometric identities test, using a larger group of participants than our preliminary study and with some refinements we describe below.

### Methods and materials

#### Participants

We recruited 50 undergraduate students at Stanford University to participate in a single hour-long session in exchange either for credit in an introductory psychology class or for pay ($12). We decided in advance to stop collecting data once we reached 50 participants. We chose a round number somewhat greater than the number of participants in the pilot study to allow us to achieve somewhat tighter confidence intervals for our dependent measures. For all of our studies, the IRB approved the experimental design, and participants provided informed written consent. 60% of the participants were female. Among the 68% of participants for whom we obtained additional demographic data, the distribution of racial identity was 29% Asian American, 26% White, 18% Black or African American, 15% Multiracial, 9% Hispanic/Latino(a), and 3% Other.

#### Materials

The problems used each consisted of a probe and four choice alternatives. The probe was always an expression of the following general form:


$$\begin{array}{l} func\left(\pm \theta \pm \Delta \right) \end{array}$$

where func was either sin (sine) or cos (cosine), and θ and Δ were numeric expressions in degrees. The value of θ was drawn from the set {10, 20, 30, 40, 50, 60, 70, 80} and could be positive or negative while ±Δ could take any of the values (–180, –90, +0, +90, +180). The order of the terms within the parentheses of the probe varied so that θ could occur first or second. If the first term was positive, its sign was not displayed. The choice alternatives were always of the form:


$$\begin{array}{l} \sin\left(\text{θ}\right)\text{ OR}-\sin\left(\text{θ}\right)\text{ OR}\cos\left(\text{θ}\right)\text{ OR}-\cos\left(\text{θ}\right) \end{array}$$

where the value of θ was the same as its value in the probe. For each participant, two blocks of forty problems were generated according to a 2x2x5x2 design in which function (sin or cos), sign of θ (positive or negative), value of ±Δ, and order (θ before Δ or Δ before θ) were fully crossed. The value of θ was selected randomly and independently on each trial.

#### Procedure

Each participant was tested individually while seated in a quiet laboratory room. At the beginning of each block, participants read instructions indicating that their task was to consider the expression at the top of each display, and to choose the equivalent expression from four possible choices. It was noted that all expressions were in degrees. They were instructed to respond “quickly but still accurately” and were asked not to use paper and pen/pencil or a calculator and not to refer to any outside sources.

A single digit subtraction problem was presented as an example, followed by a block of problems. The order of trials in each block was random, and selected independently for each block. No feedback was provided. After the participant's response was recorded on each trial, the participant clicked to initiate the presentation of the next trial. Response times (from the presentation of the problem to the mouse click indicating the participant's choice response) were recorded on every trial. At the end of each block, participants produced a confidence rating (an estimated number of correct answers out of 40) and an open-ended description of how they solved the problems, with the specific instruction to describe anything they may have visualized and any rules, mnemonics or other strategies they may have used.

Additional self-reported measures were collected after completing the second block. First, participants rated on a five point scale (Not different at all, Slightly, Somewhat, Very, Extremely different) the extent to which the way they solved the second block of questions was different from the first block of questions. An open-ended response box was provided for participants to describe any changes in the way they solved the problems. Participants then estimated how recently (in years) they encountered trigonometry in school or in work (0 if current). They also estimated how many classes they had taken that involved trigonometry or required some use of trigonometric knowledge (with the instruction that this includes not only math classes, but also applications in the sciences and other areas). Participants were then asked to rate on a five point scale (Never, Rarely, Sometimes, Often, Always) how often, in solving each block of problems, they: (1) recalled an explicit rule or formula, (2) visualized the sine or cosine graphs as waves, (3) visualized sine and cosine as x and y coordinates of a circle, (4) visualized a right triangle with sine and cosine associated with sides of a triangle, (5) used a mnemonic (memorized acronym or phrase) to help remember facts about sine and cosine, or (6) used another representation or strategy. These ratings were first collected with participants instructed to consider only the first block, and were collected a second time after the participant was instructed to consider only the second block. Participants then rated how often they had used each representation in previous classes and other experiences with trigonometry, and they also rated how much they had been exposed to each representation.

Following this, 20 additional problems were used for problem-specific self-report assessment. These 20 problems included one example of every combination of function, sign of θ, and signed value of Δ, with randomly chosen values for θ and for the order of θ and Δ. Immediately after solving each problem, participants rated the extent to which they used each representation (1–6 above) on a three point scale (not at all, a little, a lot).

At the end of the study, we also asked students to solve three problems in front of the experimenter. The experimenter instructed students to talk through what they were thinking as they were thinking it, and this think-aloud protocol was recorded.^1^ The three problems shown were: cos(−50+0), cos(20+180), and sin(90 − 70).

Due to a technical error, for the first five participants, one trial was not presented in their block of problem-specific reports, and one trial presented in their first main block was mis-specified, so that block was slightly unbalanced.

### Results

#### General performance and background measures

Our findings replicate the striking finding of our preliminary study: The ability to use knowledge of very basic aspects of trigonometry that can be captured in a very small number of rules is quite poor, even among students with fairly extensive prior exposure to trigonometry at a highly selective private university. Participants reported an average of 3.8 prior classes involving trigonometry, 95% bootstrapped^2^ confidence interval (BCI)[3.2, 4.7], *SD* = 2.7, and an average of 2.5 years since last use of trigonometry, 95% BCI [2.0, 3.0], *SD* = 1.7. Yet overall performance on our trig identity problems averaged only 52%, 95% BCI [46%, 59%].^3^

**Figure 4:**
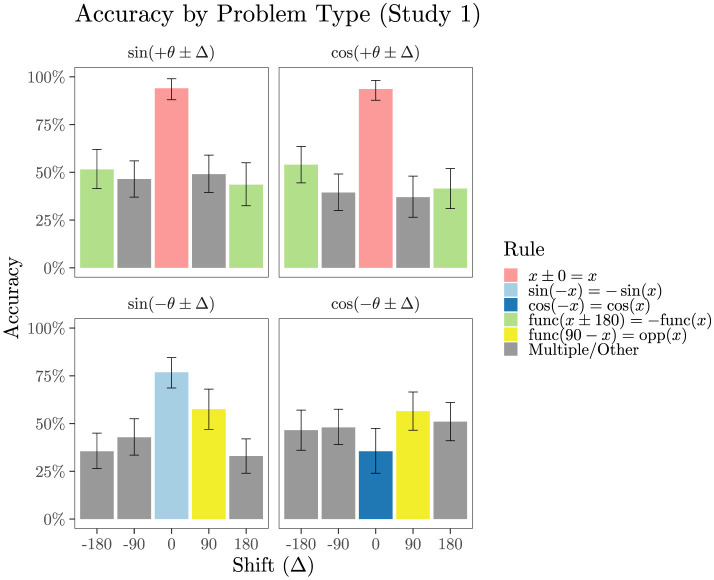
Mean accuracy (with 95% BCIs) split by problem type, from Study 1 (no lesson). The color indicates what trigonometric identity, possibly taken together with basic algebraic principles like *x*±0 = *x*, can solve each problem type. Cases labeled Multiple/Other could be solved by a combination of these trigonometric and algebraic rules. In the rules shown, func could be sin or cos; when func refers to one of these, opp refers to the other.

Figure 4 shows performance broken down by problem type, collapsing across order (θ first or Δ first) (Order had no appreciable effect on accuracy in a logistic mixed model with a random intercept and slope for each student and for each problem type, *b* = 0.04, 95% CI [−0.05, 0.12], *z* = 0.88, *p* = 0.381). As shown in pink in Figure 4, accuracy on the trivial func(θ+0) problems averaged 94% correct, 95% BCI [86%, 98%], and most (84%) participants answered all eight of these problems correctly (four with sine and four with cosine). Figure 4 also highlights eight types of problems that can be solved by the application of a single trigonometry rule, together with the rule *x*±0 = *x*. These problems are: sin(−θ+0) and cos(−θ+0); sin(θ+180), sin(θ−180), cos(θ+180) and cos(θ−180); sin(90−θ) and cos(90−θ). Mean accuracy on these problems was only 52%, 95% BCI [45, 60]. As previously observed in our pilot study, accuracy on cos(−θ+0) was quite poor, averaging 36%, 95% BCI [24, 48].

#### Self-report measures and their relationship to accuracy

Retrospective self-report ratings for blocks 1 and 2 were averaged (there were no reliable differences between blocks, -0.02, 95% CI [-0.11, 0.08], *F*(5, 294) = 0.73, *p* = 0.605). There were significantly different levels of self-reported use between representations, Kruskal-Wallis χ^2^(5) = 55.78, *p* < 0.001. With a median rating of “often” (4 on a 5 point scale), the unit circle had significantly more self-reported use than every other representation: the waves (*p* < 0.001), the right triangle (*p* = 0.018), rules/formulas (*p* = 0.005), mnemonics (*p* < 0.001), and others (*p* < 0.001) (pairwise Mann-Whitney tests corrected by Holm's procedure).

We next examined whether prior exposure to or prior use of the unit circle might explain the high degree of reliance on the unit circle in our study. A Kruskal-Wallis one-way analysis of variance showed significant variation in self-reported exposure, Kruskal-Wallis χ^2^(5) = 120.79, *p* < 0.001. Students reported significantly less exposure to mnemonics (*p* < 0.001) and “other” representations (*p* < 0.001) than the unit circle (pairwise Mann-Whitney tests corrected by Holm's procedure). However, there were no significant pairwise differences in reported exposure between the unit circle, rules, the right triangle, and waves, which all had a median rating of “quite a bit” of exposure (4 on a 5 point scale).

The self-reported ratings of prior use tell a similar story. There was significant variation in self-reported prior use, Kruskal-Wallis χ^2^(5) = 95.70, *p* < 0.001; compared to the unit circle, students reported significantly less prior use of waves (*p* = 0.003), mnemonics (*p* < 0.001) and “other” representations (*p* < 0.001). However, the median rating of prior use of the unit circle, rules, and the right triangle was “often” (4 on a 5 point scale) and there were no significant pairwise differences in reported use of these representations. Thus, the exposure ratings showed that students were taught about other representations (including rules and formulae) that they could have used, and the prior use ratings showed that many had a large amount of experience using such representations.

As a planned comparison, we examined whether reported unit circle use would be a better predictor of overall accuracy relative to other representations. We included each of the self-report representation ratings as well as classes and years since last exposure to trigonometry, as well as self-reported prior use of an exposure to the unit circle, in a logistic mixed model to predict overall accuracy (Supplementary Section 2.1). Reported unit circle use significantly accounted for independent variance after taking into account all the other predictors *b* = 0.34, 95% BCI [0.11, 0.57], *z* = 2.92, *p* = 0.004.

We next considered the relation between problem-specific ratings and performance for cos(−θ+0) and sin(−θ+0) problems. As detailed in Supplementary Section 2.2, circle use was strongly associated with correct performance on cos(−θ+0), while performance on *sin*(−θ+0) was generally accurate, independent of reliance on the unit circle. Among those reporting little or no reliance on the unit circle, the predominant error on cos(−θ+0) was −cos(θ), accounting for 75% of errors (95%BCI[61, 85]) on this problem type.

### Discussion

Study 1 finds highly consistent evidence that use of one particular visuospatial representation—the unit circle—was strongly associated with success in solving trigonometric identity problems. The unit circle was the most commonly used representation in our task, and those students who reported using it tended to have higher overall accuracy. Use of the unit circle proved to be especially strongly associated with successful performance on cos(−θ+0) problems. In spite of the simplicity of the symbolic expression used in these problems and the centrality of the cosine function in trigonometry, performance was quite poor, and the predominant response was to choose −cos(θ), rather than cos(θ), as the answer. One explanation for this error is that participants may have a tendency to follow a simple heuristic strategy which could be described as “pulling out the minus sign”—something that is consistent with symbolic rules in some situations (e.g. (−*A*) = −(*A*)), and happens to work for sin(−θ+0), but is not generally valid when dealing, as here, with a minus sign found in the argument to a function. If, on the other hand, one visualizes the cosine of a negative angle on the unit circle and compares this to the cosine of the corresponding positive angle, the identity of the corresponding values is apparent. Since no symbolic expressions are involved, there is no temptation to over-apply a “pull out the minus sign” rule.

## Study 2: comparing a formal lesson with a grounded lesson

Our findings suggest the possibility of a causal relationship between circle use and effective trigonometry performance and motivate the future exploration of such a relationship through direct manipulation of exposure to the unit circle in trigonometric reasoning. Accordingly, in Study 2, we devised two brief lessons, one grounding trigonometric relationships in the unit circle, and one promoting the use of rules without grounding them in any visuospatial conceptual structure. If, indeed, reliance on the unit circle facilitates solving trigonometric identities, then the grounded lesson should lead to more robust improvements than the formal lesson.

Note that our lessons were very brief, and the participants in our population have had extensive prior exposure to the relevant concepts. Thus, we think of these brief lessons as serving more as reminders of content previously learned than as the full source of students' knowledge of the relationships covered in these materials. We will return to this point below.

### Methods and materials

#### Participants

We recruited 70 Stanford undergraduate students to participate in a single hour-long session in exchange either for credit in an introductory psychology class or for pay ($12). 50% of the participants were female. Among the 74% of participants for whom we obtained additional demographic data, the distribution of racial identity was 31% Asian American, 25% White, 15% Black or African American, 12% Hispanic/Latino(a), 12% Multiracial, and 6% Other.

#### Procedure and design

The procedure was identical to that of Study 1, except that participants received either a grounded lesson or a formal lesson between blocks 1 and 2 of the experiment. With the addition of the lessons, we did not include a think-aloud protocol at the end of study, in order to keep the whole study within time constraints.

#### Grounded and formal lessons

The lessons were constructed to provide exposure to a parallel sequence of lesson elements, with each element presented on a single computer screen.^4^ One lesson presented each relationship in the context of the unit circle and the other presented it in the context of symbolic rules and manipulations of symbolic expressions. The set of lesson elements consisted of two subsets. The first subset dealt with the arguments to the sin and cos functions, including compound expressions, such as (70+90). In the grounded lesson, this expression was presented as describing a sequence of angular rotations of a radial line shown on an accompanying diagram, in which one component corresponded to a “special angle” (90) along with another “arbitrary” angle, in this case 70. It was noted that rotations performed in either order produce the same result, so that expressions such as (70+90) were equivalent to expressions such as (90+70). In the formal lesson, this expression was presented as an instance of an expression involving an arbitrary angle that could be represented in a rule with a variable (*x*) along with a special angle, so that (70+90) could be seen as an instance of the expression (*x*+90). It was noted that principles of ordinary arithmetic apply to such expressions, so that a rule involving (*x*+90) applies equally to expressions like (70+90) and expressions like (90+70).

The second subset of lesson elements covered eight of the 20 trigonometric identities included in our trigonometric identities test. The eight consisted of four pairs: sin(*x*+0) = sin(*x*) and cos(*x*+0) = cos(*x*); sin(−*x*+0) = −sin(*x*) and cos(−*x*+0) = cos(*x*); sin(*x*+90) = cos(*x*) and cos(*x*+90) = −sin(*x*); sin(*x*+180) = −sin(*x*) and cos(*x*+180) = −cos(*x*). From a formal, algebraic point of view, the set of rules together with principles of ordinary arithmetic were sufficient to solve all of the identity problems, although in some cases, more than one rule had to be applied to obtain the correct answer. For example, for the problem cos(−40+−180), the correct response, −cos(40), can be obtained by first applying the rule cos(−*x*) = cos(*x*) to obtain cos(40+180), then apply the rule cos(*x*+180) = −cos(*x*) to obtain −cos(40).

Each of the identities was presented in the context of a specific problem, such as cos(50+0). For the formal lesson, a rule was introduced such as cos(*x*+0) = cos(*x*). The participant was then required to apply the rule to the given expression to derive the equivalent simplified expression, in this case cos(50), then choose this expression from the simplified-expression alternatives, in this case sin(50), −sin(50), cos(50), −cos(50). Participants could not move to the next screen until the correct expression was selected. For the grounded lesson, the principle captured by the rule was introduced by depicting the given expression as a radial line on a unit circle and the corresponding projection of that line on the horizontal axis (for cosine) or the vertical axis (for sin). In both cases the general principle was stated, and participants were required to apply the principle to the given problem to select the correct answer before moving on to the next lesson element.

At the end of each subset of lesson elements, participants rated their prior familiarity, understanding, and expected ability to apply the material to problems like those encountered in the first block. To encourage productive thinking, participants in both groups then saw a final screen, stating that the rules (formal lesson) or relationships (grounded lesson) they had seen would apply directly to some of the problems they saw in the first part of the experiment, and would see again in the next part. They were also told that the rules or relationships would be helpful with other problems as well, but might need to be adapted or extended to address all of the problems. Formal lesson participants were told that “other rules from arithmetic and simple algebra might help you deal with some of the cases”, while grounded lesson participants were told, “if you can visualize the given angle as a point on the unit circle and then visualize its x or y co-ordinate, and if you can do the same with the alternative answers, this will help you solve the problem.”

### Results

#### General performance and background measures

Overall performance on the 80 problems in blocks 1 and 2 averaged 57% (95% BCI [51, 62]). Accuracy on the trivial func(θ+0) problems averaged 89% correct (95% BCI [84, 92]), and the majority (66%) of participants answered all eight of these problems correctly. Participants reported an average of 4.6 prior trigonometry classes (95% BCI [3.9, 6.1], *SD* = 4.2), and an average of 2.4 years since last use of trigonometry (95% BCI [1.9, 2.9], *SD* = 2.0). We compared the two lessons in several ways using participant ratings and other measures (Supplementary Section 2.3). There were no significant differences in the rated familiarity, understanding, or ability to apply between lessons. Students also rated the extent to which they changed their strategy or use of representations from block 1 to block 2. Students in both lesson conditions reported significantly more change in representation use relative to students with no lesson in Study 1, but there was no significant difference in the the amount of change between the formal and grounded lesson groups.

#### Effect of lessons on accuracy

We used a logistic mixed model to assess the effect of the grounded and formal lessons on accuracy, using the no-lesson participants from Study 1 as a no-lesson comparison group (Supplementary Section 2.4).

While both lessons did help students (relative to no lesson), the grounded lesson helped students achieve higher accuracy than the formal lesson. The interaction between block and lesson vs no lesson was significant (*b* = 0.06, 95% CI [0.01, 0.12], *z* = 2.28, *p* = 0.023). While there was some improvement from block 1 to block 2 even without a lesson (*b* = 0.39, 95% CI [0.14, 0.64], *z* = 3.07, *p* = 0.002), the improvement was greater for those who did receive a lesson (*b* = 0.76, 95% CI [0.11, 1.41], *z* = 2.28, *p* = 0.023). The interaction between block and lesson type was significant (*b* = 0.12, 95% CI [0.01, 0.22], *z* = 2.21, *p* = 0.027), indicating that the grounded lesson produced significantly stronger improvement than the formal lesson (*b* = 0.48, 95% CI [0.05, 0.90], *z* = 2.21, *p* = 0.027). Both groups improved significantly in accuracy from block 1 to block 2, with the grounded group showing greater improvement (*b* = 0.53, 95% CI [0.23, 0.83], *z* = 3.48, *p* = 0.001 for the formal group vs. *b* = 1.01, 95% CI [0.70, 1.31], *z* = 6.51, *p* < 0.001 for the grounded group).

**Figure 5:**
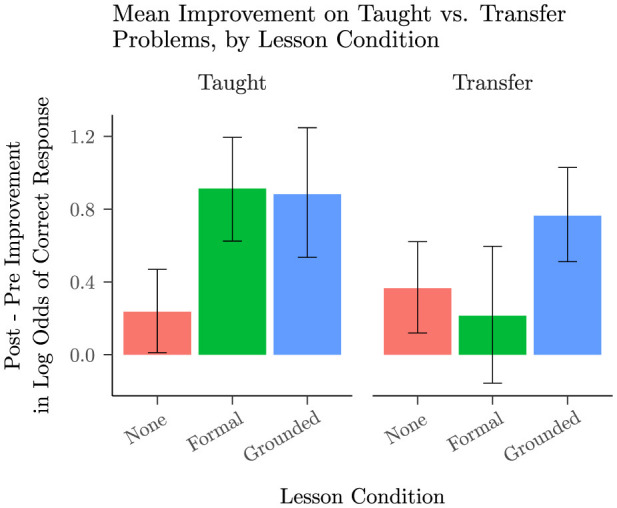
Mean improvement from block 1 to block 2 in log odds of responding correctly (with 95% BCIs) on taught vs. transfer problems, split by lesson condition: no lesson from Study 1, formal lesson from Study 2, and grounded lesson from Study 2. Although eight problems were included in the lessons, the identities sin(*x*+0) = sin(*x*) and cos(*x*+0) = cos(*x*) were expected to be trivial, and empirically most students achieved near ceiling performance before the lesson. We therefore excluded them, treating sin(−*x*+0) = −sin(*x*),cos(−*x*+0) = cos(*x*), sin(*x*+90) = cos(*x*), cos(*x*+90) = −sin(*x*), sin(*x*+180) = −sin(*x*) and cos(*x*+180) = −cos(*x*) as the taught problems in this analysis. The 12 remaining problem types were held out as transfer problems: func(*x*−180), func(−*x*+180), func(−*x*−180), func(*x*−90), func(−*x*+90), and func(−*x*−90), where func can be sin or cos.

Compared to the no-lesson baseline, we found that the formal lesson led only to enhanced performance on problem types that were included in the lesson, whereas the grounded lesson led to improvements both on taught problem types and on problem types that were held out of the lesson to assess transfer. Figure 5 shows the mean improvement for each lesson condition, broken down by whether the problem was a taught problem or a transfer problem. We extended the logistic mixed model to encompass the taught vs transfer comparison, and found these key effects: For taught problems, the effectiveness of the formal lesson (*b* = 1.24, 95% CI [0.83, 1.64], *z* = 6.01, *p* < 0.001) and the effectiveness of the grounded lesson (*b* = 1.20, 95% CI [0.81, 1.60], *z* = 5.94, *p* < 0.001) were not significantly different (*b* = −0.04, 95% CI [−0.59, 0.51], *z* = −0.13, *p* = 0.898). However, the grounded lesson showed significantly greater improvement than the formal lesson on transfer problems (*b* = 0.72, 95% CI [0.22, 1.21], *z* = 2.84, *p* = 0.004). Students who saw the formal lesson failed to show any significant improvement on transfer problems (*b* = 0.21, 95% CI [−0.14, 0.56], *z* = 1.19, *p* = 0.234) while students who saw the grounded lesson showed quite strong improvement on transfer problems (*b* = 0.93, 95% CI [0.58, 1.28], *z* = 5.16, *p* < 0.001). See Supplementary material for additional statistical details.

#### Effect of lessons on representation use

**Figure 6:**
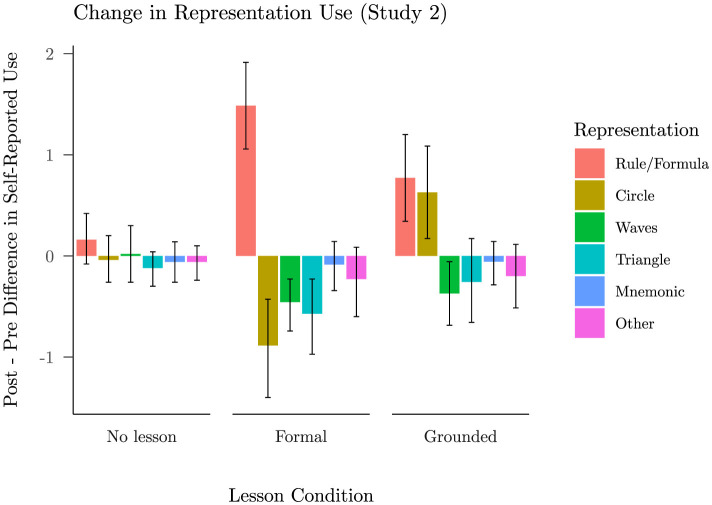
Mean change in self-reported use of each representation (with 95% BCIs), split by lesson condition: no lesson from Study 1, formal lesson from Study 2, and grounded lesson from Study 2. This change is measured as the difference between the block 1 ratings and block 2 ratings, each on a five point scale from “Never” to “Always.”

How might our grounded lesson have supported transfer to problems not explicitly taught? As discussed more fully below, we hypothesized that the unit circle might allow students to use a visualization strategy based on the unit circle to solve trigonometric identity problems. To test this, we examined changes in participants' self-reported use of different representations after participating in the grounded or the formal lesson (Figure 6), noting that, as a baseline, we found no reliable strategy changes between blocks in the no lesson condition from Study 1. As expected, students who saw the formal lesson reported an increase in rule use from block 1 to block 2, *t*_(34)_ = 6.27, *p <* 0.001, and decreased self-reported use of the unit circle, *t*_(34)_ = −3.52, *p* = 0.001. However, students who saw the grounded lesson did not simply report an increase in their use of the unit circle and a decrease in their use of rules. Instead, they reported increased use of both the unit circle, *t*_(34)_ = 2.48, *p* = 0.018, and of rules or formulae, *t*_(34)_ = 3.43, *p* = 0.002. Across participants there was no significant correlation in the reported amount of increase in circle and rule use, *r* = −0.13, 95% CI [−0.45, 0.21], *t*_(33)_ = −0.77, *p* = 0.448. Therefore, while there is variability between subjects in co-occurrence of strategies, the data did not seem to arise solely from a group that increased reliance on the unit circle and another group that increased reliance on rules. This led us to design a third study to examine alternative ways of understanding how our grounded lesson might have supported generalization beyond the problems explicitly taught.

### Discussion

In Study 2, we found that grounding trigonometric relationships in the unit circle promoted a more generalizable understanding than presenting these relationships simply in formal terms. That is, the grounded lesson improved accuracy not only on problems taught in the lesson, but also on problems held out for transfer – something the formal rule based lesson failed to do. From a practical point of view, this finding seems of central importance as scientists attempt to formulate clearly for practitioners what is needed in education to support productive transfer.

It therefore seems important to understand why our grounded lesson led to this outcome. Initially, we hypothesized that our grounded lesson would lead to increased reliance on the unit circle as a procedure for deriving representations of the quantities involved, providing a productive procedure that could be applied not only to taught but also to transfer problems, similar to the procedure Wertheimer (1959) taught young students to allow them to extend their ability to find the area of a parallelogram to figures with quite different shapes. Based on this, we expected the grounded lesson to result in increased reliance on the unit circle, at the expense of reliance on rules. However, this is not exactly what we found. The grounded lesson led to an increase in reported reliance on the unit circle and the use of rules. Our third experiment was motivated by the goal of understanding this finding and its implications for successful transfer in more detail.

## Study 3: how does the grounded lesson support transfer?

We begin by asking why our grounded lesson led to an increase in the use of rules and the unit circle. One possibility is that the grounded lesson could change the nature of the rule-like representations students used. This possibility is interesting, since such rules could potentially be more useful for performance on transfer problems that the rules taught in our formal lesson. This could occur if grounding in the unit circle changes the way a student represents the relationship expressed in a formal rule. For instance, after the grounded lesson, the quantity *x*+180 in the rule sin(*x*+180) = −sin(*x*) might be encoded as representing a position half way around the unit circle from the position of *x*, and the rule itself might be encoded as expressing the idea that when a point is rotated halfway around the circle from a starting position *x*, its vertical position will be offset from the horizontal axis by the same distance in the opposite direction, so that the value of its y coordinate will have the same magnitude but the opposite sign. This could facilitate generalization or transfer to problems in which +180 is replaced with −180 if the expression *x*−180 is also coded as corresponding to a position halfway around the circle from *x*.

There is, however, a simpler account for the increased use of both rules and the unit circle after the grounded lesson. Perhaps participants in the grounded condition relied on the taught rules, in more or less the same form as participants in the formal lesson, on taught problems, but used the approach of mapping to visualizable quantities on transfer problems. Because our self-report measure from Study 2 only asked for an overall rating of rule or circle use, the findings are consistent with this possibility.

To investigate more fully how the grounded lesson facilitated transfer, our next experiment collected problem-specific ratings of representation use. The hypothesis that participants relied predominantly on rules for taught problems but use visualization for transfer problems predicts that we would see increased rule use ratings on taught problems and increased circle use ratings on transfer problems. Alternatively, if the grounded lesson led participants to rely on grounded rather than formal rules, they might report simultaneous use of both the unit circle and rules, regardless of whether the problems were taught or transfer problems. Furthermore, if the pattern of representation use varies between participants and/or within a participant between problems, we may gain insight by asking which pattern of representation use coincided with the highest accuracy on taught problems and on transfer problems.

Our design of the current study included several additional features intended to help us understand more about the role of the grounded lesson in promoting productive reasoning. To go beyond a simple quantitative rating of representation use, we recorded participants as they thought aloud to solve four selected problems after the end of the structured experimental session. Our analysis of these recordings provided strong support for the idea that our participants frequently used grounded rules. In addition, a subset of the problems were taught to some participants and held out as transfer problems for others, with assignment to taught vs transfer counterbalanced. The study also included a manipulation of whether problems were presented with a specific angle, as in the previous studies, or with the variable θ. This allowed us to examine the possibility that participants might rely on visualization of the specific quantities involved when given a specific value, and might rely on rules (grounded or otherwise) when given a variable not corresponding to a specific value.

### Methods and materials

#### Participants

We recruited 69 Stanford undergraduates to participate in a single hour-long session in exchange either for credit in an introductory psychology class or for pay ($15 Amazon gift card). Two participants who did not complete the study within an hour were excluded (we offered them the chance to leave after an hour and they accepted). Three additional participants were excluded due to experimenter error in initializing the study, resulting in an incomplete or incorrect lesson. Of the 64 participants who completed the study, 62.5% were female. Also, among all participants who completed the study, the distribution of racial identity was 38% White, 27% Asian American, 17% Multiracial, 11% Black or African American, 5% Hispanic/Latino(a), and 3% Other.

All participants gave informed consent prior to participation. The experiment included 4 problems at the end that were solved in front of the experimenter, and in gathering consent, we explained that recording was entirely optional. Two students declined to be recorded.

#### Procedure

The procedure was very similar to the grounded condition of Study 2. Participants saw trigonometric expressions [e.g., sin(−70+180)] and tried to identify which of four simpler expressions was equivalent to the given expression. There was one block of 40 problems, followed by a brief lesson grounding these relationships in the unit circle, followed by another block of 40 problems.

There were two key differences from previous studies. First, some of the problems were selected as counterbalanced taught/transfer problems. We kept sine and cosine problems with θ+0 and −θ+0 arguments as always taught, and we kept problems with −θ+180, −θ−180, θ−90, and −θ−90 arguments as always transfer problems. The counterbalanced problems involved sine and cosine problems with θ+180, θ−180, θ+90, and 90−θ arguments. Students were randomly assigned to be taught problems with θ+180 or θ−180 arguments, and were also randomly assigned to be taught problems with θ+90 or 90−θ arguments. This 2x2 design resulted in four different versions of the grounded lesson.

The second key difference was the addition of a third block of problems during which we obtained problem-specific self-reports of strategies. These 20 problems included one example of every combination of function, sign of θ, and signed value of Δ, with randomly chosen values for θ and for the order of θ and Δ. Immediately after solving each problem, participants rated how confident they were that their answer to that problem was correct on a three point scale (not at all, somewhat, very). They then rated the extent to which they used each of the representations considered in studies 1 and 2 on a three point scale (not at all, a little, a lot).

Additionally, half of the problems used in this block included the generic variable θ instead of a specific angle measure (e.g., 20°), counterbalancing this factor with other problem type variables. Within each group receiving each version of the grounded lesson, half the students saw the generic θ problems before the instantiated angle problems, while the other half saw the instantiated angle problems before the generic θ problems.

At the end of the study, we also asked students to solve four problems in front of the experimenter. The four problems shown were: cos(−50+0), cos(20+180), sin(90 − 70), and cos(θ−180). The experimenter instructed students to talk through what they were thinking as they were thinking it. The think-aloud protocol for the 62 participants whose protocols were recorded were later transcribed for use in the analyses we report below.

### Results

#### General performance and background measures

Accuracy on the 40 problems in block 1 averaged 53%, 95% BCI [48, 59], *SD* = 0.22. Accuracy on the trivial func(θ+0) problems averaged 91% correct (95% BCI [84, 95]), and most participants (78%) answered all eight of these problems correctly. Participants reported an average of 5.5 prior classes (95% BCI [4.6, 6.6], *SD* = 4.0) and an average of 2.3 years since last use of trigonometry (95% BCI [1.8, 2.8], *SD* = 2.0).

#### Transfer effects

**Figure 7:**
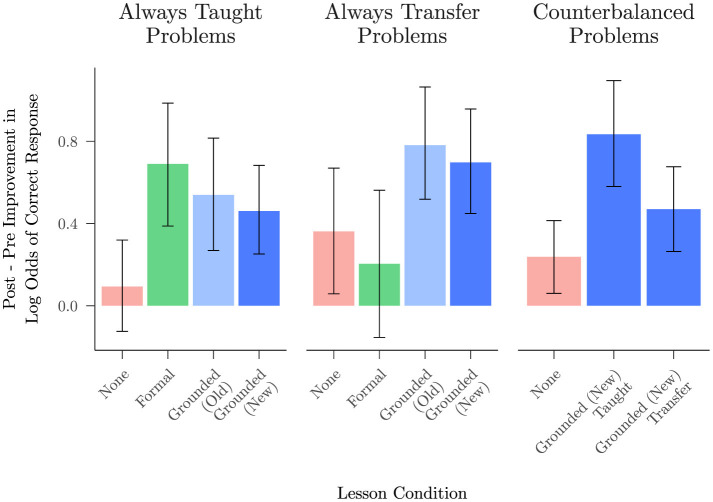
Mean improvement in log odds of responding correctly (with 95% BCIs) on taught vs. transfer problems, split by lesson condition of all studies: no lesson from Study 1, formal lesson and grounded (old) lesson from Study 2, and grounded (new) lesson from Study 3. After excluding the trivial func(θ+0) problems, the only problems always taught in the lesson were the func(−θ+0) problems. The problems always held out for transfer were func(−θ+180), func(−θ−180), func(θ−90), and func(−θ−90). The counterbalanced problems that were taught or held out for transfer in Study 3 were func(θ+180), func(θ−180), func(θ+90), and func(−θ+90). Data from the no lesson condition corresponds to the same problem types as the other conditions in each cell.

We replicated Study 2's finding that the grounded lesson facilitates transfer of a student's understanding to problems not included in the lesson materials. Figure 7 presents post-lesson improvement scores for problems that were always taught, problems that were always held out for transfer, and counterbalanced problems that were taught or held out for transfer. For always taught and always transfer problems, improvement scores on the same problems from Studies 1 and 2 are presented for comparison. Considering first the always transfer problems, we contrasted the performance improvement on these problems in Studies 2 and 3 (where the grounded lesson occurred between blocks 1 and 2) with the improvement for the same problems in Study 1 where there was no lesson, and found that the participants who saw the grounded lesson from either study improved significantly more than participants with no lesson (*b* = 0.96, 95% CI [0.15, 1.78], *z* = 2.33, *p* = 0.020). In contrast, participants receiving the formal lesson in study 2 did not improve more than participants with no lesson on these problems (*b* = −0.20, 95% CI [−0.71, 0.31], *z* = −0.77, *p* = 0.440). Though the improvement on problems always held out for transfer was slightly larger after the grounded lesson in Study 2 than it was in Study 3, the difference was not significant (*b* = −0.13, 95% CI [−0.62, 0.35], *z* = −0.54, *p* = 0.587).

Focusing next on the results from the current study on the counterbalanced problems, we sought to assess whether students showed improvement after the lesson on these problems, with a particular interest in improvement scores when these problems were held out for transfer. We used a logistic mixed model to predict whether each trial was answered correctly (Supplementary Section 2.5). Students improved significantly from block 1 to block 2 in overall accuracy, *b* = 0.69, 95% CI [0.52, 0.87], *z* = 7.72, *p* < 0.001, and the interaction between block and transfer was significant, *b* = −0.32, 95% CI [−0.57, −0.07], *z* = −2.55, *p* = 0.011. Examining simple effects among the contributing conditions, we found that within the first block, there was no significant difference in accuracy between taught and transfer problems, *b* = 0.10, 95% CI [−0.15, 0.35], *z* = 0.79, *p* = 0.430, but within the second block, students solved more taught problems correctly than transfer problems, *b* = −0.36, 95% CI [−0.65, −0.07], *z* = −2.40, *p* = 0.016. Crucially, students did still show significant improvement on transfer problems from block 1 to block 2, *b* = 0.47, 95% CI [0.26, 0.67], *z* = 4.42, *p* < 0.001.

An exploratory analysis to understand the reduced transfer effect observed with the counterbalanced stimuli (Supplementary Section 2.6) considered the potential influence of the specific problem type on both baseline problem success and transfer, using results obtained from Study 3 as well as the previous two studies. This analysis showed that our grounded lesson produced a robust transfer effect among variants of problems involving a shift of ±180. The grounded lessons led to transfer across all problem variants with a shift of ±180, while the formal lesson did not. In contrast, the pattern of transfer after a grounded lesson among variants of problems involving a shift of ±90 was mixed. Our tentative interpretation of the full pattern of findings with such problems is that grounding in the unit circle interacted with students' prior understanding of problems involving cos(90−θ) and sin(90−θ). These are among the easiest of the problems in our data set (Figure 4), and students' reports of their strategies on such problems often grounded them in relationships within a right triangle, where they have a very clear grounded interpretation (Supplementary Section 2.7). Nevertheless, there were some signs of positive transfer among problems involving ±90. A further finding to emerge from this analysis was the observation that, both before and after receiving either a grounded or a formal lesson, students had less success with problems including cos(−θ), regardless of the value of the shift (0, ±90 or ±180), suggesting that their tendency to rely on the heuristic of “pulling out the minus sign” was not completely dispelled by either type of lesson.

#### Overall rule and circle use

Study 3 replicated the finding that the grounded lesson led to an increase in self-reported overall use of both the unit circle, *t*_(63)_ = 1.74, *p* = 0.088, and rules and formulae, *t*_(63)_ = 3.99, *p* < 0.001. As in Study 2, we did not find evidence of the students falling into two distinct types, one reporting extensive rule/formula use but little circle use and the other reporting extensive circle use but little rule/formula use. Instead, we found that the relationship between a student's mean reliance on the unit circle and mean reliance on a rule or formula was not significantly different from 0, *r* = −0.15, 95% CI [−0.38, 0.10], *t*_(62)_ = −1.18, *p* = 0.242. Furthermore, 61% of participants reported a mean reliance on both the unit circle and a rule or formula greater than or equal to 1.5.

#### Problem specific rule and circle use ratings

Next we turned to results from trials in which participants provided problem specific ratings, focusing on the counterbalanced problems that control for problem type across participants. The results from this block corroborate the impression that our participants often relied jointly on rules and the unit circle in this block of trials, which occurred after they all received our grounded lesson. In support of this, the mean reported reliance on a rule or formula was 2.13, 95% BCI [1.96, 2.29], while mean reported reliance on the unit circle was 1.98, 95% BCI [1.81, 2.16]. Of the participants who reported relying a little or a lot on the unit circle or on a rule or formula for a particular problem, 42% reported relying a little or a lot on the other representation during the same trial.

**Figure 8:**
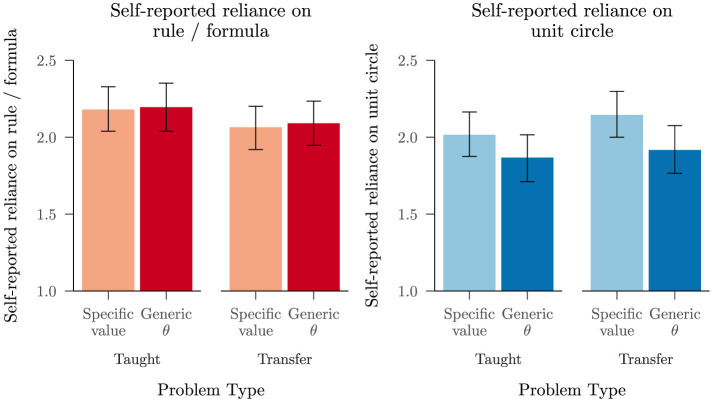
Mean self-reported reliance (with 95% BCIs) on a rule or formula and on the unit circle, split by taught vs. transfer problem and by specific value vs. generic θ, using the block of problem-specific ratings from Study 3.

If the increase in rule and circle use that we observe after the grounded lesson occurred because participants used rules on taught problems and rely on the unit circle on transfer problems, we would expect to see higher ratings of rule use on taught problems and higher ratings of circle use on transfer problems. As shown in Figure 8, participants reported using rules and the unit circle across both problem types, with only a slight tendency for rule or circle use to vary as a function of whether a problem was taught or held out for transfer. A cumulative link mixed model for reliance on rules (including a random intercept for each participant and random effects of transfer vs. taught, value type, and their interaction) found slightly greater reliance on rules for taught than for transfer problems, *b* = −0.27, 95% CI [−0.51, −0.02], *z* = −2.11, *p* = 0.035 and no other significant effects. A corresponding analysis for reliance on the unit circle did not find a significant effect of taught vs. transfer. Instead, it found slightly more reliance on the circle for problems involving a specific angle than for problems with a generic θ, *b* = −0.48, 95% CI [−0.74, −0.22], *z* = −3.58, *p* < 0.001, as shown in the right panel of Figure 8.

#### Problem specific rule and circle use ratings and accuracy

**Figure 9:**
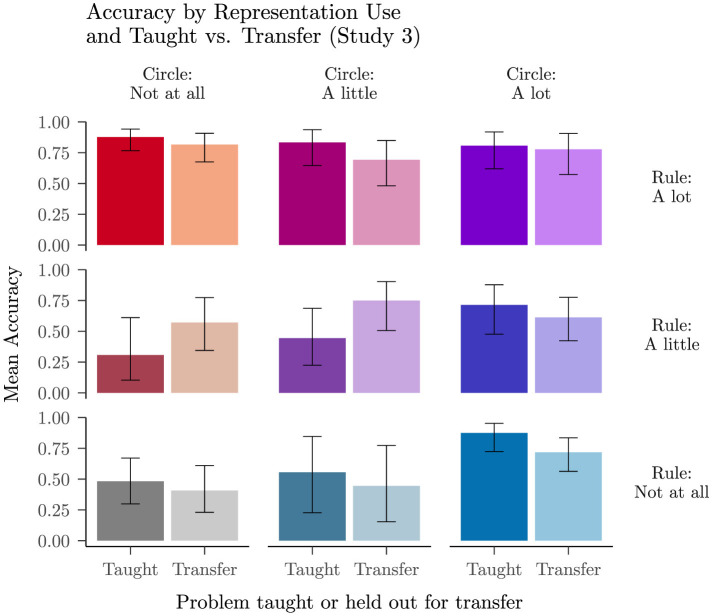
Accuracy on trials in Study 3 for each combination of self-reported reliance on the unit circle and on a rule/formula, split by whether the problem was taught in the lesson or held out as a novel transfer problem. The results are taken the problems that were counterbalanced as taught or transfer.

The problem-specific ratings block also allows us to examine how the representation a student reported using on each problem relates to their accuracy in answering the problem. For this analysis, we focus on students' performance on the counterbalanced problems (Figure 9), using accuracy on these problems in block 3, the block with problem-specific reports, as the dependent measure. The analysis revealed that when students reported using neither the circle or a rule/formula, they performed poorly, but when students reported using the unit circle or a rule/formula or both on a particular trial, they were more likely to answer correctly. We used a logistic mixed model to predict accuracy, with self-reported specific use of the unit circle, self-reported specific use of a rule or formula, and transfer (vs. taught) as predictors, along with their interactions (Supplementary Section 2.8). The analysis revealed a significant main effect of reported rule/formula use, *b* = 0.80, 95% CI [0.32, 1.27], *z* = 3.28, *p* = 0.001, a non-significant trend toward a main effect of reported circle use, and a significant interaction of reported use of the unit circle and reported use of a rule or formula, *b* = −0.87, 95% CI [−1.47, −0.27], *z* = −2.86, *p* = 0.004. On trials in which a student reported not relying on the unit circle, higher rating of rule or formula use significantly predicted better accuracy, *b* = 1.65, 95% CI [0.80, 2.51], *z* = 3.79, *p* < 0.001. On trials in which a student reported relying a lot on the unit circle, there was no significant effect of self-rated rule or formula use, *b* = −0.09, 95% CI [−0.74, 0.57], *z* = −0.26, *p* = 0.798.

#### Protocol evidence for use of grounded rules

The findings considered so far support the view that students who saw the grounded lesson tended to report increased use of both rules and the unit circle. This finding has led us to the view that an important function of the grounded lesson is to allow students to appreciate the rules of trigonometry, not just as arbitrary arrangements of symbols, but as expressions capturing meaningful visuospatial relationships. Specifically, we suggest that the grounded lesson may facilitate students encoding and expressing rules in terms of the unit circle, which may in turn support both good performance on taught problems as well as transfer to problems not explicitly taught. Evidence from the think-aloud protocols of students' solutions of the problem cos(20+180) provided additional support for this view (we provide the full transcripts of these protocols in Supplementary Section 3; transcripts of students protocols for the other three problems are available in our OSF repository https://osf.io/3dtp9/).

When students encountered the cos(20+180) problem during the problem specific rating block, 13 of the 64 participants selected the option “a lot” in rating their reliance on a rule or formula and selected “not at all” in rating their reliance on the unit circle. Our reading of the think-aloud protocols for these participants suggested to us that some of their rules were grounded in the unit circle. Participant #25 fell in this group for problem-specific rating of this particular problem, and was also the only participant in their general ratings to report always using a rule or formula and never using any other representation. However, in the think-aloud protocol this student expressed an understanding in terms of visuospatial transformation and resulting relationships, using gesture as well as words: “I know it's going to go basically shift right across that [rotates hand] … it's just pretty much negative of whatever the other angle is …”.

To examine this group's tendency to ground rules in the unit circle, we looked at language used in the think-aloud protocol for each of the 13 participants in this subset. Two participants (#22, #62) explicitly mentioned using a “circle”, and an additional six participants (#3, #14, #16, #25, #54, #57) mentioned some combination of “visualizing”, “rotating”, “angles”, and “quadrants”. Two participants (#42, #58) mentioned visualizing angles on the x-y plane in their general strategy description, but not in this particular think-aloud protocol. One participant (#36) mentioned a wave-based “graph” visualization. Only two participants (#10, #19) mentioned no visualization.

In summary, the think-aloud protocol for many of the participants who reported relying on rules and not on the unit circle appeared to include references to visualization and the unit circle. Although they differed in how they described their strategy both in problem-specific and in more global ratings, the preponderance of successful participants in Study 3 appear to have relied at least in part on the unit circle or rules grounded in the unit circle. This is consistent with the view that the grounded lesson may have facilitated encoding and expressing rules in terms of the unit circle. We explore this idea more fully below.

## General discussion

The findings from the three studies reported here show that trigonometric relationships provide a powerful example of how a coherent, visuospatially grounded, conceptual structure can make symbolic expressions meaningful, allowing them to be applied and generalized successfully. In Study 1, we observed how students approach solving trigonometric identity problems, and found that students most often reported using the unit circle. Students who reported using the unit circle also tended to have higher accuracy. Other students, who did not report using the unit circle or reported using it less often, were more likely to rely on faulty heuristics like “pulling out the minus sign”, leading to errors on cos(−θ) = −cos(θ). Study 2 randomly assigned students to a brief lesson, either based strictly on formal rules or grounded in the unit circle, allowing us to test the causal nature of the relationship observed in Study 1. This study found that both lessons led to improvements on relationships explicitly taught, but only the grounded lesson lead to enhanced performance on transfer problems not explicitly taught in the lesson.

Next, we sought to examine how the unit circle taught in the grounded lesson supported transfer to untaught problems. Study 3 counterbalanced the assignment of some problems to either be taught in the lesson or held out for transfer. We replicated Study 2's finding that the grounded lesson facilitated transfer of a student's understanding to problems not included in the lesson, though the effect was restricted to a subset of the counterbalanced problems. Study 3 also included a block of problems where students reported their use of rules and formulas, the unit circle, and other strategies. Students reported relying slightly more on a rule or formula when solving taught problems than transfer problems. However, across all problem types, students often reported relying on both the unit circle and a rule or formula on a particular problem. At the end of Study 3, participants also completed a brief think-aloud protocol, which revealed that most of the successful participants, even those who self-reported rule use with no circle use, relied at least in part on rules grounded in the unit circle.

Across our studies, we found that grounding trigonometric relationships in the unit circle facilitated successful problem solving and generalization. We now consider two different ways our grounded lesson might have facilitated this outcome.

First, the grounded lesson may provide a a general procedure, sufficient to solve both the taught and transfer problems in our studies. Specifically, our unit circle lesson might lead students to use the numbers inside the parentheses of a trigonometric expression to map the expression to a position on an internally represented, schematic unit circle, and then to use the cosine or sine function to project that position onto the *x* or *y* axis through the center of the circle to determine the sign and magnitude of the quantity represented by the expression. They could apply this same procedure both to the probe expression and to the candidate choice alternatives to find the candidate that has the same sign and magnitude as the probe expression.

Alternatively, the unit circle may help students represent mathematical rules, not simply as abstract symbolic expressions, but as relationships between entities and their properties in structured spatial relationships. Treating rules as representing such relationships can also support systematic generalization beyond specific taught relationships. For example, our unit circle based lesson can help students think of the expression cos(*x*+180) = −cos(*x*) as expressing the idea that a point located halfway around the unit circle from another point is the same distance from the *y* axis as the original point, but in the opposite direction. If the student knows that 180 degree rotations in either direction, denoted by offsets of ±180 degrees, will take a point half way around the circle, then the rule will apply equally to cos(*x*−180) as to cos(*x*+180).

It seems likely that there is some partial validity to both of these possibilities, and the relative importance of each may be difficult to pinpoint exactly. While the first was the one we started with after Study 1, our findings across Studies 2 and 3 led us to attribute increased importance to the second possibility. The key finding that began to point us toward the idea of grounded rules was our finding in study 2 that the grounded lesson led to increased reports both of rule use and of use of the unit circle in problem solving. Further consideration of participants reports of rule and circle use in study 3, including qualitative and quantitative analyses of participant's recorded protocols as they solved the cos(20+180) problem corroborated this interpretation further: even those who said they only used rules expressed their understanding in terms of words and gestures consistent with grounding these rules in the unit circle.

### The unit circle as an integrated conceptual structure that may be challenging to learn

Whether by providing a general procedure or by grounding rules, the unit circle exhibits several of the characteristics that Case et al. (1996) identified in what he called central conceptual structures. We call these structures integrated conceptual structures, to emphasize that they depend on linking symbolic and structural elements as well as linking two different ways of thinking about positions of points on the plane.

To bring out this linkage, we briefly revisit the identities


sin(-θ)=-sin(θ),

cos(-θ)=cos(θ),

and


sin(θ+180)=-sin(θ),

cos(θ+180)=-cos(θ).

Seen through the lens of purely symbolic expressions, these are arbitrary and confusing expressions. Why, for example, should the same relationship hold for sin and cos in one case but not in the other? In the first pair, why is it that sin and not cos has a minus sign on the right hand side?

To make sense of these identities—to see why they hold, rather than as arbitrary expressions to be memorized—one must understand the symbols involved through the lens of their meaning in the visuospatial model of the unit circle. Quite a lot is in play here. One must be certain of that sin picks out the vertical coordinate of the point at the circular position θ, while cos picks out the horizontal coordinate, and not the other way around. The validity of the relationship also depends on treating θ and −θ as referencing positions reached by equal and opposite rotations from the correct reference point; if one treated the intersection of the circle with the positive end of the vertical axis as 0 the relationship would no longer hold. Thus, the elements of the expressions require a very specific coordination of symbols and numbers with the spatial model for the two identities to make sense. For the second pair of identities, 180 must be seen as corresponding to a rotation half way around the circle, thereby placing the point reached after the rotation exactly opposite the point at θ so that it is clear that it must be same distance in the opposite direction from the point at θ in both the horizontal and the vertical directions. For students who have a firm grasp of these alignments, the spatial model supports an intuitive understanding of the relationships expressed in both of these pairs of identities.

These observations may help explain why only a subset of the students in our study successfully relied on the unit circle and why only a subset of those who did not do so spontaneously showed a benefit from our brief grounded lesson. As we have noted, this lesson introduces the relevant concepts and relationships very briefly, and so it may be better to think of it as a reminder or refresher, rather than as a sufficient basis for internalizing and integrating all of the conventions involved to be able to use them effectively after the brief reminder lesson. The reminder lesson may not “stick” if the conventions are unfamiliar. For example, if the student does not already know that 180 degrees corresponds to a rotation half way around the circle, the brief engagement with this fact in our grounded lesson may not be sufficient for them to engage with it when they see the expression cos(θ+180) in a test problem after the lesson.

### Toward a curriculum that produces robust grounded learning

The findings of these studies provided a springboard for asking whether a focus on grounding trigonometric relationships in the unit circle could be used as the basis for a curriculum that could lead to robust learning of these relationships in students without prior background in trigonometry beyond exposure to the basic trigonometric concepts typically encountered in high-school geometry classes. To this end, we obtained permission to observe the teaching of trigonometry in two pre-calculus classrooms at a public high school near Stanford, and we invited students from these classrooms to go through the protocol of Study 2 in the unit-circle condition, as they were completing a six-week unit on trigonometry. Strikingly, though their textbook and classroom instruction included exposure to the unit circle and to the trigonometric identities covered in our lesson, all but one of the students who chose to participate performed near chance on the pre-test portion of the study, and performance on the post-text did not show a statistically reliable improvement. This led us to begin to appreciate the challenges many students face when attempting to engage with this subject matter, and to undertake a multi-year effort to develop a grounded curriculum that begins to address some of these challenges, helping more students achieve success, as described in the sequel to this article (Mickey et al., 2025).

## Conclusion

Across our studies, the unit circle revealed itself to be a strong, coherent conceptual structure, creating a fertile ground for learning and understanding trigonometry through the interplay of visuospatial and rule-based approaches. While the unit circle could serve as a standalone procedure for solving problems, and it could also be used alongside independent rules or formulae, we have argued that successful students learn and use rules that are more intrinsically tied to the unit circle. Students can learn how to map parts of a symbolic expression onto meaningful properties of the unit circle, and this grounded understanding may facilitate application and generalization of rules in order to solve problems successfully. Continuing this research by addressing remaining theoretical questions and by working closely with educators to facilitate implementation will have important benefits, both for our understanding of the role of grounding symbolic expressions in visuospatial representations, and also for helping students learn mathematics more effectively. Our own efforts in this direction are described in Mickey et al. (2025).

## Data Availability

The datasets presented in this study can be found in online repositories. The names of the repository/repositories and accession number(s) can be found at: https://osf.io/3dtp9/: Making math more meaningful by grounding rules in a coherent conceptual structure.

## Footnotes

^1^In gathering consent, we explained that recording was entirely optional. One student declined to be recorded.

^2^We used 10,000 bootstrap runs to resample subjects, and confidence intervals have 95% point-wise coverage and are bias corrected and accelerated (BCa) (Efron, 1987).

^3^Supplementary Section 1 provides information about gender effects in Studies 1–3. In brief, male participants outperformed females in Study 1 and prior to the lessons in Studies 2 and 3. The main patterns of findings we report in the main text showed the same pattern across genders, but were slightly stronger in women, perhaps because more men performed at ceiling accuracy levels.

^4^Complete lesson materials used in Studies 2 and 3 are available in our OSF repository at https://osf.io/dkng9.
