# Supplementary material for: Grounding mathematics in an integrated conceptual structure, part I: experimental evidence that grounded rules support transfer that formal rules do not
Source: Front Psychol. 2025 Jun 3;16:1507670. doi: 10.3389/fpsyg.2025.1507670 (PMC12170660; doi:10.3389/fpsyg.2025.1507670)
Supplement: Supplementary file 1 [file Data_Sheet_1.pdf]

# Supplementary Materials for:

# Grounding Mathematics in a Coherent Conceptual Structure, Part I: Grounded Rules Support Transfer that Formal Rules Do Not

Mickey, K. W. & McClelland, J. L.[1]
Stanford University, Stanford, CA, USA

[1] Corresponding author: jlmcc@stanford.edu

# 1 GENDER-RELATED EFFECTS IN STUDIES 1-3

## 1.1 Study 1

Of the 50 participants in Study 1, 60% of the participants were female.

First, we examined the relationship between gender and accuracy. Male participants (M=64%) answered significantly more problems correctly than female participants (M=43.5%), $b = 0.51$, 95% CI [0.21, 0.80], $z = 3.31$, $p = .001$. We also examined the relationship between gender and background variables. There was no significant difference between male and female participants in terms of number of relevant classes, $b = 0.55$, 95% CI $[-1.01, 2.12]$, $t(47) = 0.71$, $p = .479$, or in terms of years since last exposure to trigonometry, $b = -0.31$, 95% CI $[-1.33, 0.71]$, $t(48) = -0.61$, $p = .546$.

We also looked at the relationship between gender and self-reported use of the unit circle. Male participants (M=3.05) did not report significantly different circle use than female participants reported (M=3.4), using a Mann-Whitney test, $W = 260.00$, $p = .424$. Male and female participants also did not report significantly different prior use of the unit circle, $W = 287.00$, $p = .792$, or exposure to the unit circle, $W = 284.50$, $p = .751$.

Next, we asked whether gender moderated the effect of circle use on accuracy. The interaction between gender and circle use on accuracy was not significant, $b = 0.20$, 95% CI $[-0.07, 0.48]$, $z = 1.44$, $p = .149$. That is, men's relationship between circle use and accuracy, $b = 0.66$, 95% CI [0.27, 1.05], was not significantly stronger than the women's relationship, $b = 0.25$, 95% CI $[-0.12, 0.62]$. Across circle use, men did have significantly higher accuracy than women, $b = 0.49$, 95% CI [0.23, 0.75], $z = 3.65$, $p < .001$.

Even with prior use and exposure to the unit circle included in the model, the interaction between gender and circle use on accuracy was also not significant, $b = 0.19$, 95% CI $[-0.08, 0.45]$, $z = 1.40$, $p = .163$. That is, men's relationship between circle use and accuracy, $b = 0.80$, 95% CI [0.38, 1.22], was not significantly stronger the women's relationship, $b = 0.43$, 95% CI [0.03, 0.82].

Finally, we investigated gender in the context of the relationship observed between problem-specific ratings and performance for $\cos(-\theta + 0)$ and $\sin(-\theta + 0)$ problems. The three-way interaction between gender, problem-specific circle use, and function was not significant, $b = 0.78$, 95% CI $[-1.26, 2.82]$, $z = 0.75$, $p = .455$. Neither the two-way interaction between gender and problem-specific circle use, $b = 0.37$, 95% CI $[-1.13, 1.86]$, $z = 0.48$, $p = .630$, nor the two-way interaction between gender and function were significant, $b = 0.16$, 95% CI $[-1.76, 2.09]$, $z = 0.17$, $p = .868$. Across circle use and across function, the difference between men and women in accuracy on these problems was not significant, $b = 0.90$, 95% CI $[-0.85, 2.65]$, $z = 1.00$, $p = .315$.

## 1.2 Study 2

Of the 70 participants in Study 2, 50% of the participants were female. We randomly assigned participants to lesson conditions. 17 of the 35 participants assigned to the formal rule-based lesson were female. 18 of the 35 participants assigned to the lesson grounded in the circle were female.

First, we examined the simple effect of gender on accuracy within block 1 of Study 2, prior to the lesson materials. Male participants (M=56%) answered significantly more problems correctly than female participants (M=37%), $b = 3.48$, 95% CI [1.17, 5.80], $z = 2.95$, $p = .003$.

Next, we asked whether gender moderated the effect of the lesson on accuracy. To predict whether each trial was answered correctly, we used a logistic mixed model with block, lesson condition, gender, and their interactions as predictors. We included a random intercept for each participant, as well as a random effect of block.

We included data from both Studies 1 and 2, coding condition with two contrasts: general lesson effect (both lessons vs. no lesson), and lesson type (grounded lesson vs. formal lesson). The three-way interaction

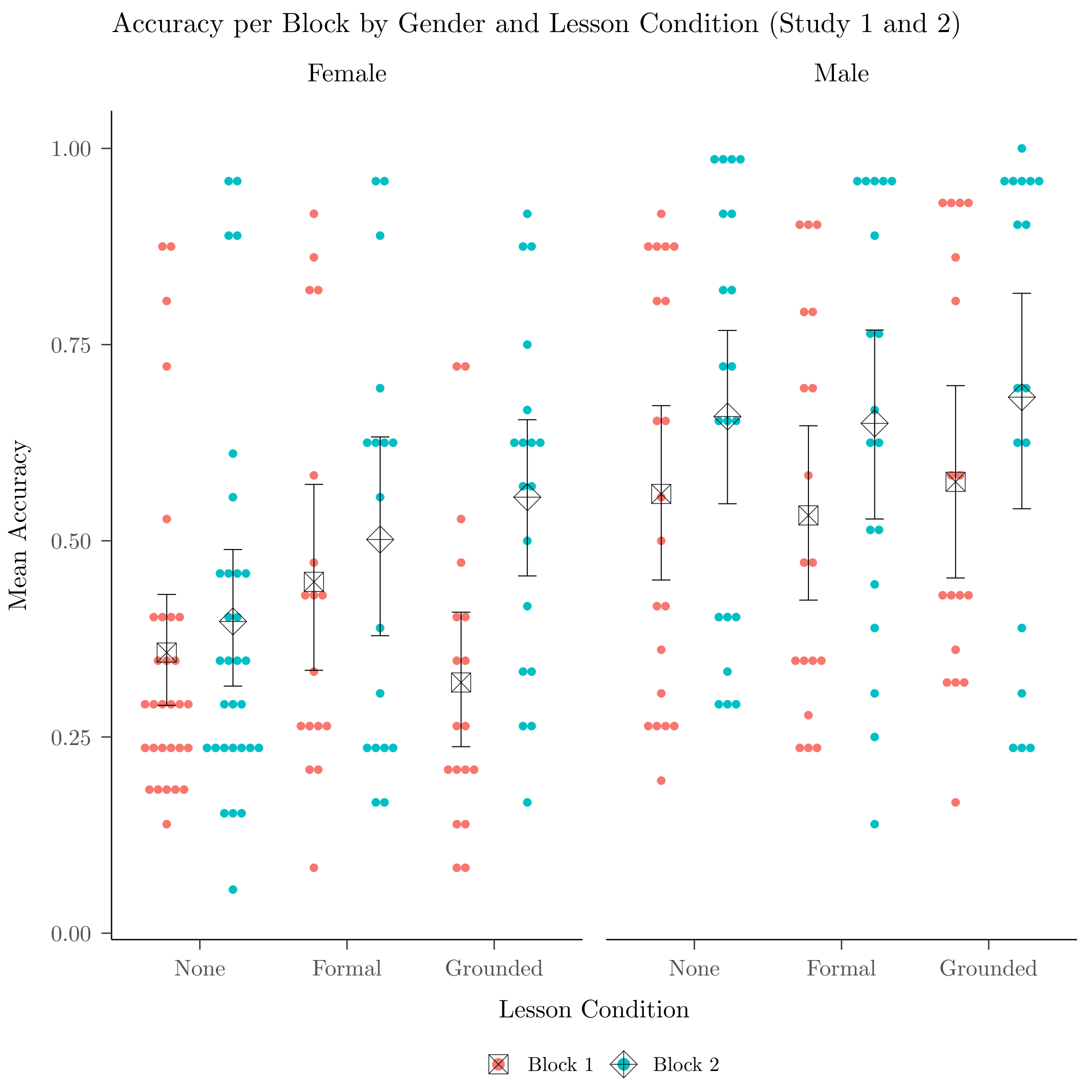


**Figure S1.** Mean accuracy, by lesson condition and gender, split by block

between gender, block, and lesson type was marginally significant, $b = -0.10$, 95% CI $[-0.20, 0.00]$, $z = -1.88$, $p = .060$. See Figure S1 for the breakdown of the data into the conditions contributing to this analysis. For women, the interaction between block and lesson type was significant, $b = 0.85$, 95% CI $[0.28, 1.42]$, $z = 2.93$, $p = .003$, while for men it was not significant, $b = 0.06$, 95% CI $[-0.54, 0.65]$, $z = 0.19$, $p = .846$. That is, women showed marginally greater improvement after the grounded lesson relative to the formal lesson than men did. This may, however, have been partially due to a ceiling effect in men, with the test unable to distinguish improvement among high performing participants.

Next, we considered the interactions of gender in a model that also compares taught versus transfer problems. Neither the four-way interaction between gender, block, transfer and general lesson effect nor the four-way interaction between gender, block, transfer, and lesson type were significant. We assessed several three-way interactions involving gender, all of which were not significant: between gender, block, and transfer, between gender, block and general lesson effect, between gender, block and lesson type, between gender, transfer and general lesson effect, and between gender, transfer and lesson type.

## 1.3 Study 3

Of the 64 participants in Study 3, 62.5% of the participants were female.

First, we examined the simple effect of gender within block 1 of Study 3, prior to the lesson materials. Male participants (M=64%) answered significantly more problems correctly than female participants (M=49%), $b = 0.62$, 95% CI [0.11, 1.12], $z = 2.39$, $p = .017$. We also examined whether gender moderated the effect of accuracy across all problems, and this interaction was not significant, $b = 0.11$, 95% CI [$-0.03$, 0.25], $z = 1.55$, $p = .122$.

Next, we investigated whether there was an interaction of gender with block and transfer type in Study 3, using counterbalanced problems only. The three-way interaction between gender, block and taught versus transfer was not significant. Nor were the two-way interactions between gender and transfer or between gender and block.

We also considered the interactions of gender in a model that also includes problem type. We analyzed problem type with three orthogonal contrasts: the shift effect of $\pm 180$ vs 90, the difference between +180 and -180, and the difference between a positive and negative angle $\theta$ with a shift of 90. The four-way interaction between gender, block, taught versus transfer, and shift (90 versus 180) was significant, $b = 0.59$, 95% CI [0.12, 1.06], $z = 2.44$, $p = .015$. For men, the three-way interaction between block, shift (90 versus 180), and taught versus transfer was significant, $b = 2.02$, 95% CI [0.46, 3.59], $z = 2.53$, $p = .011$, while for women it was not significant, $b = -0.32$, 95% CI [$-1.36$, 0.72], $z = -0.60$, $p = .548$. That is, men showed greater improvement on 180 problems relative to 90 problems more often on transfer than on taught problems, whereas women showed greater improvement on 180 problems relative to 90 problems across both taught and transfer problems. Again, men may have been disporportionally affected by a ceiling effect, with the test unable to distinguish improvement among high performing participants. Supplemental Appendix B explores why transfer may have been stronger for some problems over other problems, by considering specific characteristics of different problems. Further research that incorporates gender into such analyses would help to build explanations for some of the potential interactions observed in our studies that involve gender.

Additionally, we explored the interaction of gender with problem-specific self-reported use of different representations. One model predicted problem-specific use of the unit circle, and another model predicted problem-specific use of rules. For predicting circle use, the three-way interaction between gender, transfer (versus taught) and value type (specific angle versus generic theta) was not significant. The two-way interaction between gender and value type was not significant, but the two-way interaction between gender and transfer was significant, $b = -0.30$, 95% CI [$-0.57$, $-0.04$], $z = -2.26$, $p = .024$. Women reported significantly more circle use on transfer problems than on taught problems, $b = 0.84$, 95% CI [0.21, 1.47], $z = 2.62$, $p = .009$, whereas men showed no significant difference in circle use between transfer and taught problems, $b = -0.37$, 95% CI [$-1.23$, 0.48], $z = -0.86$, $p = .390$. For predicting rule use, the three-way interaction between gender, transfer and value type was not significant. Neither was the two-way interaction between gender and value type. The two-way interaction between gender and transfer was marginally significant, $b = 0.25$, 95% CI [$-0.02$, 0.53], $z = 1.84$, $p = .065$. Women reported significantly less rule use on transfer problems than on taught problems,$b = -0.89$, 95% CI [$-1.53$, $-0.24$], $z = -2.70$, $p = .007$, whereas men showed no significant difference in rule use between transfer and taught problems, $b = 0.13$, 95% CI [$-0.74$, 1.00], $z = 0.29$, $p = .769$. Further work is required to disambiguate possible relationships, and to measure potential interactions between gender and strategies on accuracy.

Table S1. Parameters for a logistic mixed model using self-reported measures of representation use and previous experience to predict whether each trial was answered correctly in Study 1 (no lesson).

| | Simple model | | | Model incl. UC experience | | |
|---|---|---|---|---|---|---|
| Predictor | $b$ | 95% CI | $p$ | $b$ | 95% CI | $p$ |
| (Intercept) | -0.73 | [-2.00, 0.55] | .266 | 0.67 | [-1.05, 2.40] | .446 |
| Rule / formula use | 0.08 | [-0.27, 0.43] | .652 | 0.04 | [-0.29, 0.37] | .817 |
| Unit circle use | 0.24* | [0.05, 0.43] | .013 | 0.34** | [0.11, 0.57] | .004 |
| Waves use | -0.22 | [-0.51, 0.07] | .139 | -0.26† | [-0.54, 0.02] | .066 |
| Right triangle use | 0.22† | [-0.03, 0.46] | .079 | 0.23† | [0.00, 0.46] | .054 |
| Mnemonic use | -0.22 | [-0.61, 0.16] | .256 | -0.41† | [-0.84, 0.02] | .060 |
| Other representation use | -0.11 | [-0.38, 0.17] | .442 | -0.11 | [-0.37, 0.15] | .399 |
| N of relevant classes | 0.11 | [-0.02, 0.24] | .107 | 0.10 | [-0.02, 0.23] | .109 |
| Years since last trig | -0.03 | [-0.21, 0.15] | .725 | -0.05 | [-0.22, 0.12] | .554 |
| Prior unit circle use | | | | 0.16 | [-0.30, 0.62] | .496 |
| Exposure to unit circle | | | | -0.46* | [-0.91, -0.01] | .045 |

# 2 EXTENDED DATA ANALYSES FROM STUDIES 1-3

## 2.1 Accuracy by Circle Use in Study 1

As a planned comparison, we examined whether reported unit circle use would be a better predictor of overall accuracy relative to other representations. We first considered a model that included each of the self-report representation ratings as well as classes and years since last exposure in a logistic mixed model to predict overall accuracy. Table S1 (Simple model columns) shows the fitted parameters of this model. Reported unit circle use significantly accounted for independent variance after taking into account all the other predictors. The relationship is statistically reliable $b = 0.24$, 95% CI [0.05, 0.43], $z = 2.47$, $p = .013$. Of the other self-report measures, only the right triangle predictor was marginally significant, $b = 0.22$, 95% CI $[-0.03, 0.46]$, $z = 1.76$, $p = .079$. After adding to our model self-reported prior use of and exposure to the unit circle (last two columns of table), the unit circle remained the only significant predictor among the representation use ratings, $b = 0.34$, 95% CI $[0.11, 0.57]$, $z = 2.92$, $p = .004$. Exposure to the unit circle was negatively related to accuracy, $b = -0.46$, 95% CI $[-0.91, -0.01]$, $z = -2.00$, $p = .045$. Figure S2 presents a scatterplot showing the independent prediction of overall accuracy from self-reported use of the unit circle in a logistic regression model, controlling for self-reported use of each representation, number of classes, years since last exposure, self-reported prior use of the unit circle and exposure to the unit circle.

Further analysis suggests that many of those who reported using the right triangle might have used a representation with many of the properties of the unit circle. Of the eleven subjects who reported using the right triangle often or always six explicitly described their mental representation as a circle in their open-ended responses, and three other subjects described both a coordinate plane or axes and negative angles or quadrants. Thus, for most subjects who reported using the right triangle, their success may be explained in part by actual use of the unit circle or their use of a systematic representation of angles and trigonometric relationships on the (x,y) coordinate plane that is functionally similar to the unit circle.

## 2.2 Performance on $cos(-\theta)$ by reported circle use in Study 1

Figure S3 shows the distribution of responses to $\cos(-\theta + 0)$ and $\sin(-\theta + 0)$ problems, broken down by a student's problem-specific self-reported use of the unit circle. Looking only at these types of problems,

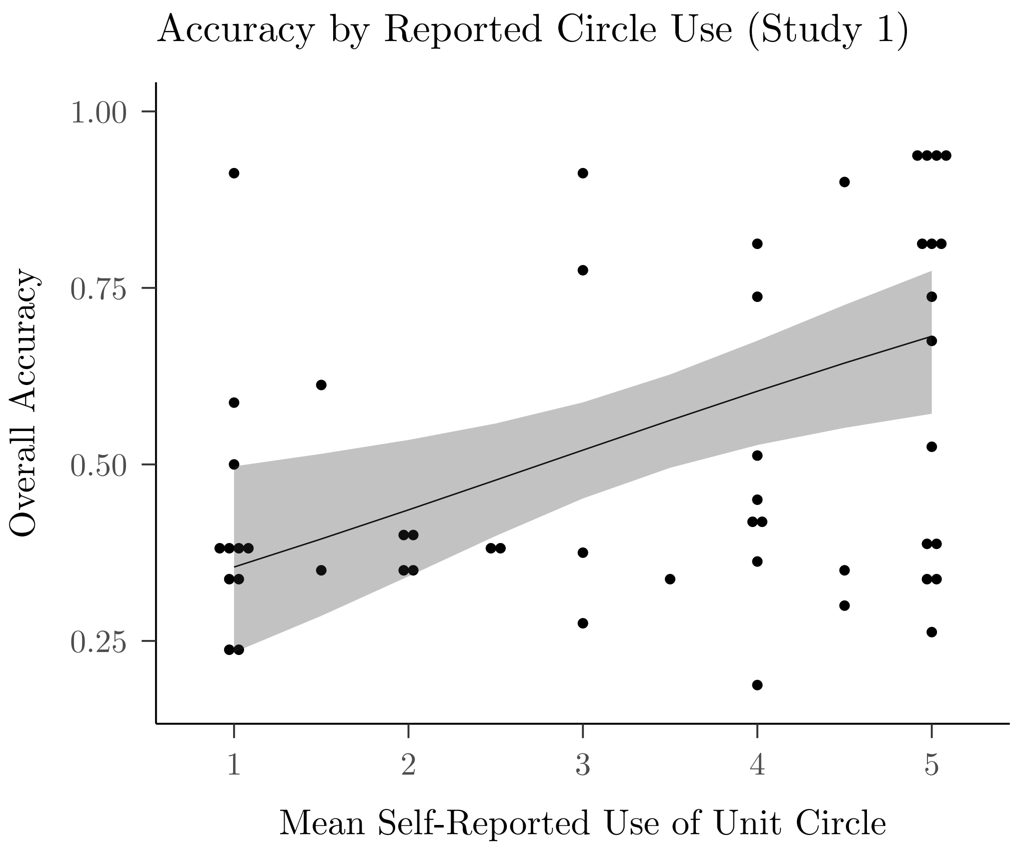


**Figure S2.** Mean accuracy by self-reported use of the unit circle (averaged across blocks 1 and 2) in Study 1, after controlling for self-reported use of each representation, number of classes, years since last exposure, self-reported prior use of the unit circle and exposure to the unit circle. The best-fitting regression line is shown along with its 95% confidence band. Each point represents a participant, The labels for each rating level were: 1='Never', 2='Rarely', 3='Sometimes', 4='Often', 5='Always'.

we used a logistic mixed model to predict whether a student answered each problem correctly. The model included function (sine or cosine), self-reported use of the unit circle, and their interaction as fixed effects and also allowed these effects to vary between subjects. We included trials from the blocks 1 and 2 as well as the problem-specific report block, and we applied each student's circle rating to every similar problem in blocks 1 and 2. A logistic mixed model showed that the interaction between problem type (sin vs cos) and circle rating was significant, $b = -3.64$, 95% CI $[-5.98, -1.31]$, $z = -3.06$, $p = .002$. In a simple effects analysis, for the $\cos(-\theta + 0)$ problem type, the problem-specific unit circle rating predicts higher accuracy, $b = 4.62$, 95% CI $[2.56, 6.67]$, $z = 4.40$, $p < .001$, while for the $\sin(-\theta + 0)$ problems, we found no such relation, $b = 0.97$, 95% CI $[-0.53, 2.48]$, $z = 1.27$, $p = .206$.

## 2.3 Comparability of lessons in Study 2

Halfway through the lesson and again at the end of the lesson, each student rated how familiar the lesson material was, how much they understood, and how well they may be able to apply the material. Using Mann-Whitney tests corrected by Holm's procedure, there were no significant differences in the rated familiarity, understanding, or ability to apply between lessons. In the first section of both lessons, the median familiarity was 4.5 (between "Mostly" and "Completely"), the median understanding was 5 ("Completely"), and the median ability to apply was 4 ("Mostly"). In the second section of both lessons,

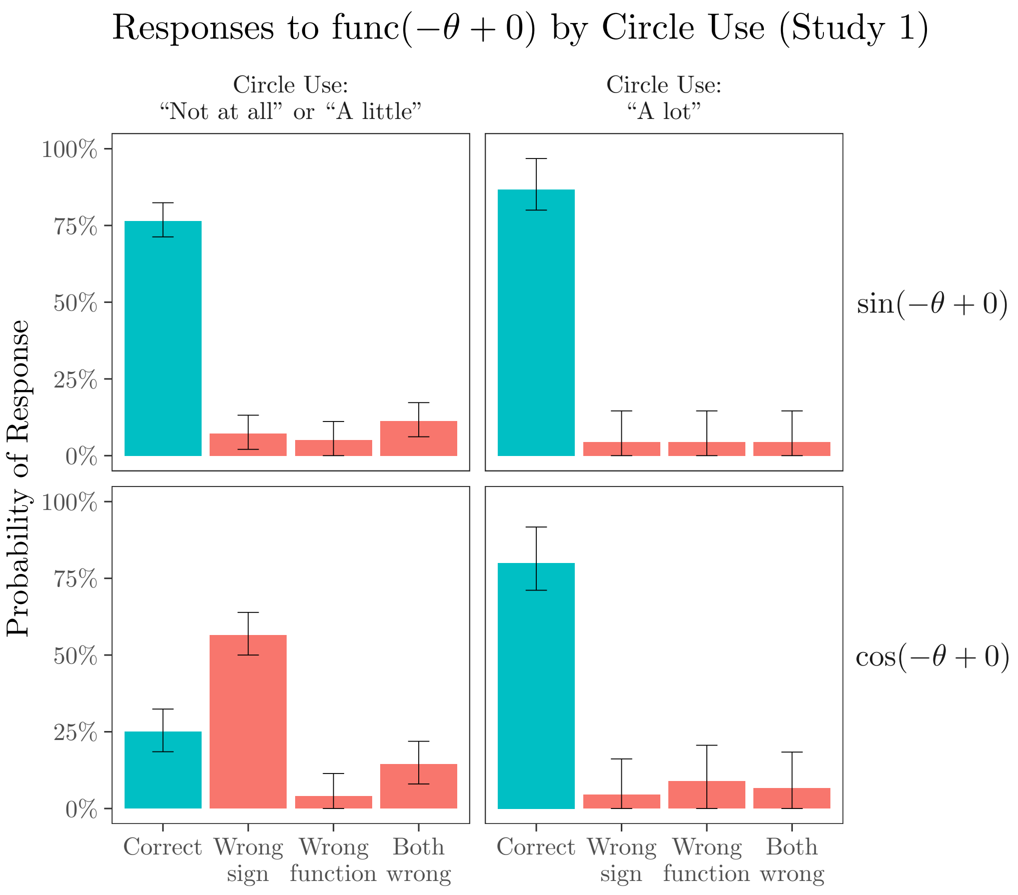


**Figure S3.** The distribution of responses to func($-\theta + 0$) in all three blocks of Study 1 with 95% simultaneous CIs, split by function and by level of unit circle use, based on problem-specific unit circle use rating produced in block 3.

the median familiarity was 3 ("Partially"), the median understanding was 4 ("Mostly"), and the median ability to apply was 4 ("Mostly").

We can also compare lessons by looking at how students rated their own confidence after solving the first block (before the lesson) and after solving the second block (after the lesson). In a one-way analysis of variance, we found that lesson condition does influence change in confidence rating, $F(2, 117) = 6.70$, $p = .002$. After block 1, students estimated how many problems they solved correctly (out of 40 problems), and the average across lesson condition was 15.23, 95% BCI [13.50, 17.13], $SD = 10.15$. Students in Study 1 with no lesson increased their estimated accuracy from block 1 to block 2 by 3.12, 95% CI [0.76, 5.48]. With contrasts comparing Study 1 (no lesson) to both lessons, students in both lessons showed significantly higher confidence than students with no lesson, $t(117) = 3.61$, $p < .001$. Students in the formal lesson increased their estimated problems solved correctly by 8.14, 95% CI [5.32, 10.97], and students in the grounded lesson increased their estimate by 9.37, 95% CI [6.55, 12.19]. With a contrast comparing the grounded lesson to the formal lesson, there was no significant difference in their effect on the change in confidence, $t(117) = 0.61$, $p = .543$.

Students rated the extent to which they changed their strategy or use of representations from block 1 to block 2 on a five point scale ("Not different at all", "Slightly", "Somewhat", "Very", "Extremely different"). Students in the first study with no lesson reported a median "slight" change, $M$ = 2.04, 95% CI [1.70, 2.38], while students in the formal lesson condition and in the grounded lesson both had a median

Table S2. Parameters for logistic mixed models using block, lesson condition (lesson (Study 2) vs. no lesson (Study 1)), lesson type (grounded vs. formal), and, for one model, transfer (vs. taught) to predict whether each trial was answered correctly.

| | Simple model | | | Model including transfer | | |
|---|---|---|---|---|---|---|
| Predictor | *b* | 95% CI | *p* | *b* | 95% CI | *p* |
| (Intercept) | 0.17 | [-0.07, 0.41] | .172 | 0.24† | [-0.01, 0.49] | .062 |
| Block | 0.32*** | [0.24, 0.40] | $<$ .001 | 0.36*** | [0.28, 0.45] | $<$ .001 |
| Transfer (vs. taught) | | | | -0.18*** | [-0.24, -0.12] | $<$ .001 |
| Lesson (vs. none) | 0.11 | [-0.05, 0.27] | .194 | 0.14 | [-0.03, 0.30] | .105 |
| Lesson type | 0.01 | [-0.31, 0.32] | .968 | -0.03 | [-0.35, 0.29] | .862 |
| Block × Transfer | | | | -0.11*** | [-0.17, -0.05] | $<$ .001 |
| Block × Lesson | 0.06* | [0.01, 0.12] | .023 | 0.08** | [0.03, 0.14] | .003 |
| Block × Lesson type | 0.12* | [0.01, 0.22] | .027 | 0.09 | [-0.02, 0.19] | .125 |
| Transfer × Lesson | | | | -0.08*** | [-0.12, -0.04] | $<$ .001 |
| Transfer × Lesson type | | | | 0.09* | [0.02, 0.17] | .017 |
| Block × Transfer × Lesson | | | | -0.06** | [-0.09, -0.02] | .003 |
| Block × Transfer × Lesson type | | | | 0.09* | [0.02, 0.17] | .011 |

self-report of changing “somewhat”, (Formal: $M$ = 3.14, 95% CI [2.74, 3.54]; Grounded: $M$ = 2.77, 95% CI [2.37, 3.17]). In a one-way analysis of variance, condition had a significant effect on rated change in representation or strategy, $F(2, 117) = 9.36$, $p < .001$. With a contrast comparing the first study (no lesson) to both lessons, students in both lessons reported significantly more change in representations relative to students with no lesson, $t(117) = 4.13$, $p < .001$, but in a contrast comparing the grounded lesson to the formal lesson, there was no significant difference, $t(117) = -1.30$, $p = .198$.

## 2.4 Effect of lessons on accuracy in Study 2

The logistic mixed model we used to assess the effects of the grounded and formal lessons used in Experiment 2 on accuracy included a random intercept for each participant, as well as a random effect of block. We included data from both Studies 1 and 2, coding condition with two contrasts: general lesson effect (both lessons vs. no lesson), and lesson type (grounded lesson vs. formal lesson). We tested the interaction of lesson condition and block. Table S2 presents this simple model’s fitted parameters.

To explore the potential interaction of lesson condition with taught versus transfer problems, we used a logistic mixed model to predict whether each trial was answered correctly. We included a random intercept for each participant, as well as a random effect of block, transfer, and their interaction. We included data from both Studies 1 and 2, coding condition with two contrasts: general lesson effect (both lessons vs. no lesson), and lesson type (grounded lesson vs. formal lesson). We tested the interaction of condition, block, and transfer. Note that because we assigned some problem types to be taught in the lessons and held out other problem types for transfer, we could still consider the contrast between taught and transfer problems in the no lesson group, in order to understand the baseline difference between different problem types.

Table S2 presents the fitted parameters of this full model, which considers taught versus transfer problems. The three-way interaction between block, transfer, and general lesson was significant, $b = -0.06$, 95% CI $[-0.09, -0.02]$, $z = -2.95$, $p = .003$. While the no lesson group did show some improvement from block 1 to block 2 on problems assigned to be taught in the lessons, $b = 0.38$, 95% CI [0.07, 0.70], $z = 2.36$, $p = .018$, both lessons showed stronger improvement on taught problems, $b = 1.67$, 95% CI [0.83, 2.51], $z = 3.89$, $p < .001$. The effectiveness of the formal lesson on taught problems, $b = 1.24$,

Table S3. Parameters for a logistic mixed model using block and transfer type to predict whether each trial was answered correctly in Study 3. This model considers only problems which were counterbalanced as taught in the lesson or held out for transfer: func($\theta + 90$) or func($90 - \theta$), and func($\theta + 180$) or func($\theta - 180$).

| Predictor | $b$ | 95% CI | $p$ |
|---|---|---|---|
| (Intercept) | 0.62*** | [ 0.30, 0.97] | $<$ .001 |
| Block | 0.69*** | [ 0.52, 0.87] | $<$ .001 |
| Transfer (vs. taught) | -0.13 | [-0.28, 0.09] | .227 |
| Block $\times$ Transfer | -0.32* | [-0.53, -0.04] | .011 |

95% CI [0.83, 1.64], $z = 6.01$, $p < .001$, and the effectiveness of the grounded lesson on taught problems, $b = 1.20$, 95% CI [0.81, 1.60], $z = 5.94$, $p < .001$, were not significantly different, $b = -0.04$, 95% CI [$-0.59$, 0.51], $z = -0.13$, $p = .898$.

The three-way interaction between block, transfer, and lesson type was also significant, $b = 0.09$, 95% CI [0.02, 0.17], $z = 2.55$, $p = .011$. Again, the no lesson group showed some limited improvement from block 1 to block 2 on problems assigned to be held out for transfer in the lesson conditions, $b = 0.40$, 95% CI [0.11, 0.69], $z = 2.72$, $p = .007$. This is comparable to the improvement on taught problems for the no lesson group, and indicates that, without a lesson of either type, the taught and transfer problems show comparable improvements with practice. Interestingly, we did not observe a significant general lesson advantage for transfer problems over no lesson, $b = 0.33$, 95% CI [$-0.43$, 1.10], $z = 0.86$, $p = .390$. However, the grounded lesson showed significantly greater improvement than the formal lesson on transfer problems, $b = 0.72$, 95% CI [0.22, 1.21], $z = 2.84$, $p = .004$. Students who saw the formal lesson failed to show any significant improvement on transfer problems, $b = 0.21$, 95% CI [$-0.14$, 0.56], $z = 1.19$, $p = .234$. This suggests that the strictly rule-based approach worked for problems where a taught rule can be directly applied, but that our participants could not extend the approach to other problems not explicitly taught. On the other hand, students who saw the grounded lesson showed quite strong improvement on transfer problems, $b = 0.93$, 95% CI [0.58, 1.28], $z = 5.16$, $p < .001$.

## 2.5 Effect of taught vs. transfer on accuracy in Study 3

The logistic model used to assess the effect of taught vs. transfer problems on accuracy in Study 3 included transfer (vs. taught), and their interaction as fixed effects. The model also included a random intercept for each participant, as well as a random effect of block, transfer, and their interaction. The model and fitted parameters are shown in Table S3.

## 2.6 Exploring Transfer by Problem Type in Study 3

In the main text, we briefly discussed a logistic mixed model that included problem type as a fixed effect, along with block, transfer, and the interactions of these effects. The data for this model included only the problems that were counterbalanced in Study 3: func($\theta + 180$), func($\theta - 180$), func($\theta + 90$), and func($90 - \theta$). We analyzed problem type with three orthogonal contrasts: the shift effect of $\pm 180$ vs 90, the difference between +180 and -180, and the difference between a positive and negative angle $\theta$ with a shift of 90. Table S4 presents the fitted parameters of this model. There was a significant block by shift interaction, $b = 0.43$, 95% CI [0.13, 0.73], $z = 2.84$, $p = .005$, and a significant transfer by shift interaction, $b = 0.32$, 95% CI [0.02, 0.62], $z = 2.09$, $p = .037$; breaking this pattern down, there was no

Table S4. Parameters for a logistic mixed model using block, transfer, and problem type to predict whether each trial was answered correctly in Study 3. This model considers only problems which were counterbalanced as taught in the lesson or held out for transfer: func$(\theta+90)$ or func$(90-\theta)$, and func$(\theta+180)$ or func$(\theta-180)$.

| Predictor | | 95% CI | *p* |
|---|---|---|---|
| (Intercept) | 0.64*** | [0.30, 0.97] | $<.001$ |
| Block | 0.70*** | [0.52, 0.87] | $<.001$ |
| Transfer (vs. taught) | -0.09 | [-0.28, 0.09] | .324 |
| Shift (90 or $\pm 180$) | 0.10 | [-0.11, 0.31] | .347 |
| 90 type ($\theta+90$ or $90-\theta$) | 0.73*** | [0.50, 0.97] | $<.001$ |
| 180 type ($\theta+180$ or $\theta-180$) | -0.21† | [-0.44, 0.02] | .080 |
| Block × Transfer | -0.28* | [-0.53, -0.04] | .025 |
| Block × Shift | 0.43** | [0.13, 0.73] | .005 |
| Block × 90 type | -0.12 | [-0.44, 0.20] | .464 |
| Block × 180 type | 0.19 | [-0.12, 0.51] | .227 |
| Transfer × Shift | 0.32* | [0.02, 0.62] | .037 |
| Transfer × 90 type | -0.15 | [-0.56, 0.25] | .455 |
| Transfer × 180 type | -0.25 | [-0.65, 0.16] | .232 |
| Block × Transfer × Shift | 0.28 | [-0.15, 0.70] | .202 |
| Block × Transfer × 90 type | -0.02 | [-0.47, 0.44] | .943 |
| Block × Transfer × 180 type | -0.29 | [-0.74, 0.16] | .213 |

significant shift effect within taught problems, $b=-0.12$, 95% CI $[-0.43, 0.19]$, $z=-0.79$, $p=.432$, but within transfer problems, students solved those with a shift of $\pm 180$ correctly more often than those with a shift of 90, $b=0.33$, 95% CI $[0.04, 0.62]$, $z=2.21$, $p=.027$. Because the lesson occurred between blocks 1 and 2, and the lesson is what determines whether a problem is taught or held out for transfer, we might expect to see a three-way interaction between block, transfer, and shift. There was a trend toward such an interaction, but it was not statistically significant, $b=0.28$, 95% CI $[-0.15, 0.70]$, $z=1.28$, $p=.202$. Motivated by these results, this supplemental appendix explores the details of the pattern of transfer for problems of different types.

#### 2.6.0.1 Transfer among 180 problems.

Problems with a shift of $\pm 180$ seem well-suited for grounding in the unit circle. Figure S4, which includes results from Studies 1, 2, and 3, shows the change in accuracy on problems with a shift of $\pm 180$, from block 1 to block 2. Each set of four bars shows performance on test problems with a specific combination of the direction (sign) of $\theta$ and the direction (sign) of the 180 degree shift; within each set of four bars, the results are further broken down by the type of $\pm 180$ problems taught during the lesson (if any), and by whether the lesson was formal or grounded. Note first that all four problem types showed a small and nearly identical improvement from block 1 to block 2 in the No Lesson condition from Study 1 (grey bars at the left of each set of four bars in the figure). Next, consider the results following the formal lesson, in which the $\theta+180$ problems were taught, as shown in the second bar from the left in each group of four bars. Here we see that formal lesson participants showed improvement from block 1 to block 2 on these problems as taught, but showed no transfer advantage over the no-lesson baseline on other variants of $\pm\theta \pm 180$ problems. In contrast, when the same problems were taught in a grounded lesson (third bar from left in each set of four bars), there was a transfer advantage in the improvement scores relative to no lesson.

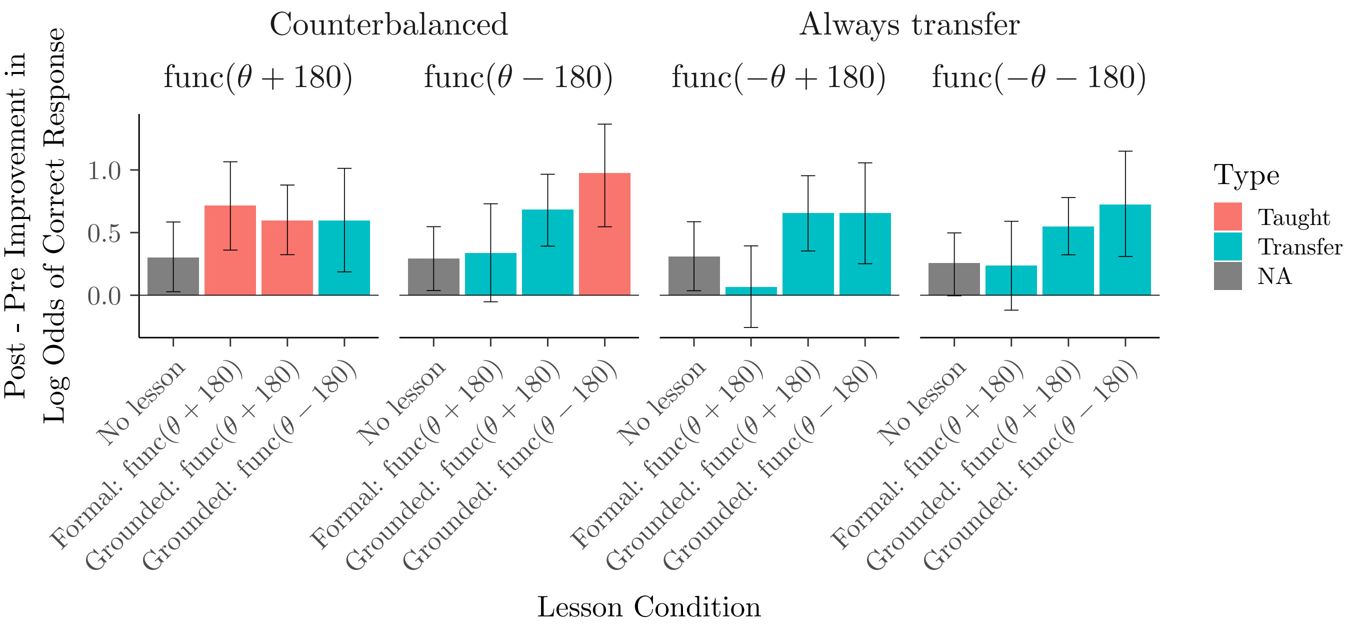


**Figure S4.** Mean improvement in log odds of responding correctly on 180 problems from block 1 to block 2 (with 95% BCIs), split by problem type and lesson condition (no lesson from Study 1, formal lesson from Study 2, and grounded lesson from Studies 2 and 3). The grounded lesson condition was split based on the type of 180 problem presented in the lesson, with the grounded lesson of Study 2 grouped together with students in Study 3 who were taught func$(\theta + 180)$.

The fourth bar from the left in each group shows improvement scores for participants who received were taught $\theta - 180$ problems in a grounded lesson. Participants did very well on such problems themselves (orange bar in the second set of four bars), and also showed a transfer advantage on each of the other type of test problem.

#### *2.6.0.2 Transfer among 90 problems.*

Figure S5 displays the change in accuracy on problems with a shift of $\pm 90$, from block 1 to block 2. Again, the formal lesson only facilitates improvement on its taught 90 problems, func$(\theta + 90)$. Unlike problems with a shift of $\pm 180$, the 90 problems that were counterbalanced showed little or no transfer. The problems that were always held out of the lesson did, however, seem to show moderate transfer. It is possible that the problems we chose to counterbalance had specific characteristics that inhibited transfer.

First, we consider why the grounded lesson seemingly failed to facilitate transfer func$(-\theta + 90)$. These problems are unique due to the knowledge that students already possess prior to the lesson. Figure 3 in the main text shows the block 1 performance for each problem type in Study 1, and we see the same five problems with the highest accuracy in the same rank order when we aggregate block 1 performance across Studies 1, 2, and 3, as reported here. Unsurprisingly, students answered the trivial func$(\theta + 0)$ problems correctly the most often: 90%, 95% BCI [86, 93], on $\cos(\theta + 0)$, and 88%, 95% BCI [83, 91], on $\sin(\theta + 0)$. The problem type with the next highest block 1 performance was $\sin(-\theta + 0)$, at 76%, 95% BCI [70, 80]. The func$(-\theta + 90)$ problems held the fourth and fifth highest block 1 performance, out of 20 problem types: 63%, 95% BCI [56, 68], on $\sin(-\theta + 90)$, and 58%, 95% BCI [52, 64], on $\cos(-\theta + 90)$.

This strong block 1 performance suggests that, in addition to having general strategies, some students retrieved specific strategies for solving func$(-\theta + 90)$. The explicit rule that students most commonly reported in the open-ended description of strategies after block 1 was func$(90 - \theta) = $ opp$(\theta)$ (or an instance thereof). Other students also reported using a triangle-based schema, consisting of complementary angles and knowledge of trigonometric functions as ratios of sides of a triangle. When the grounded

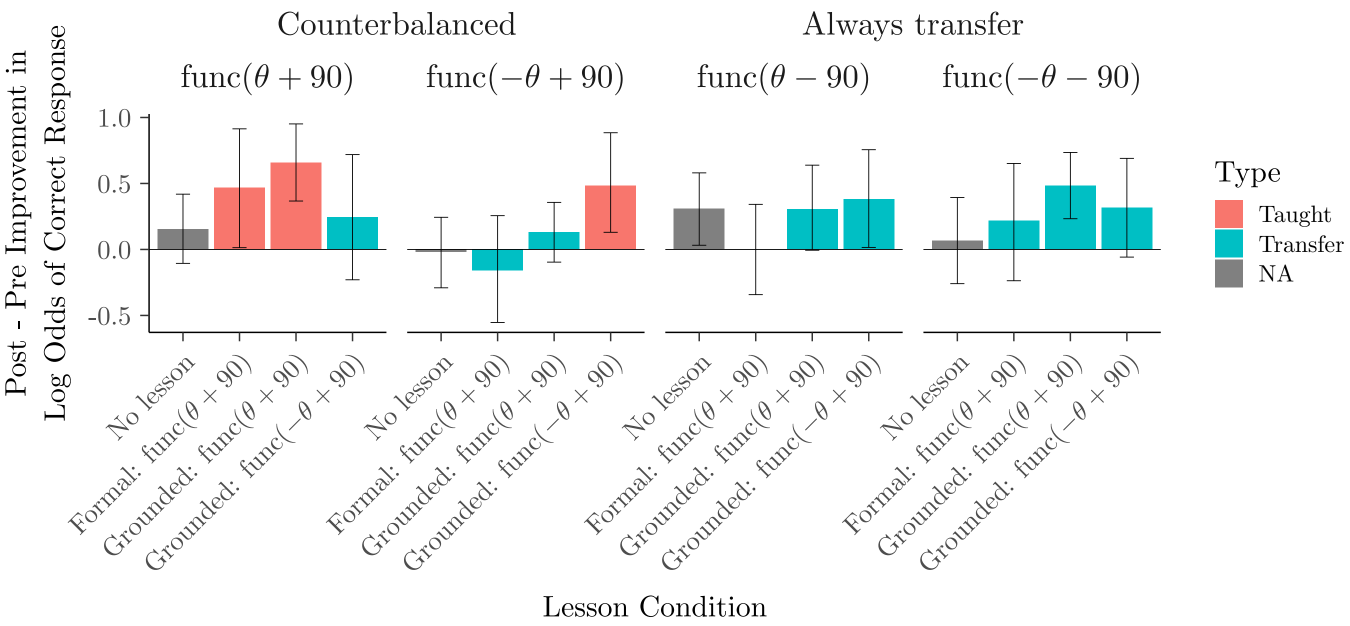


**Figure S5.** Mean improvement in log odds of responding correctly on 90 problems from block 1 to block 2 (with 95% BCIs), split by problem type and lesson condition (no lesson from Study 1, formal lesson from Study 2, and grounded lesson from Studies 2 and 3). The grounded lesson condition was split based on the type of 90 problem presented in the lesson, with the grounded lesson of Study 2 grouped together with students in Study 3 who were taught func$(\theta + 90)$.

**Table S5.** Mean accuracy and 95% CI, broken down by block and problem type, for participants who saw func$(\theta + 90)$ in the grounded lesson from Studies 2 and 3.

| | Block 1 | | Block 2 | |
|---|---|---|---|---|
| Problem type | $b$ | 95% CI | $b$ | 95% CI |
| func$(\theta - 90)$ | 0.37 | [0.29, 0.45] | 0.47 | [0.37, 0.56] |
| func$(-\theta + 90)$ | 0.60 | [0.50, 0.68] | 0.63 | [0.54, 0.71] |
| func$(-\theta - 90)$ | 0.41 | [0.33, 0.49] | 0.57 | [0.49, 0.64] |

lesson included func$(\theta + 90)$ problems and not func$(-\theta + 90)$, students may have adopted a more general approach based on the unit circle, and discarded or ignored their old strategies, which may not have seemed useful or relevant during the lesson. This scenario would result in roughly equal performance in block 2 on func$(-\theta + 90)$ and other transfer problems with a shift of 90. Because of the higher block 1 performance, though, the func$(-\theta + 90)$ problems would have little change in accuracy and therefore appear to show little or no transfer. The actual pattern of results, shown in Table S5, indeed involves greater improvement for func$(\theta - 90)$ and func$(-\theta - 90)$ than func$(-\theta + 90)$. However, this improvement was not quite enough to remove any difference in block 2. In an exploratory mixed logistic model of success on these problems on block 2, students were significantly more accurate on func$(-\theta + 90)$ than on func$(\theta - 90)$, $b = 1.02$, 95% CI [0.60, 1.44], $z = 4.75$, $p < .001$, or on func$(-\theta - 90)$, $b = 0.61$, 95% CI [0.20, 1.02], $z = 2.89$, $p = .004$.

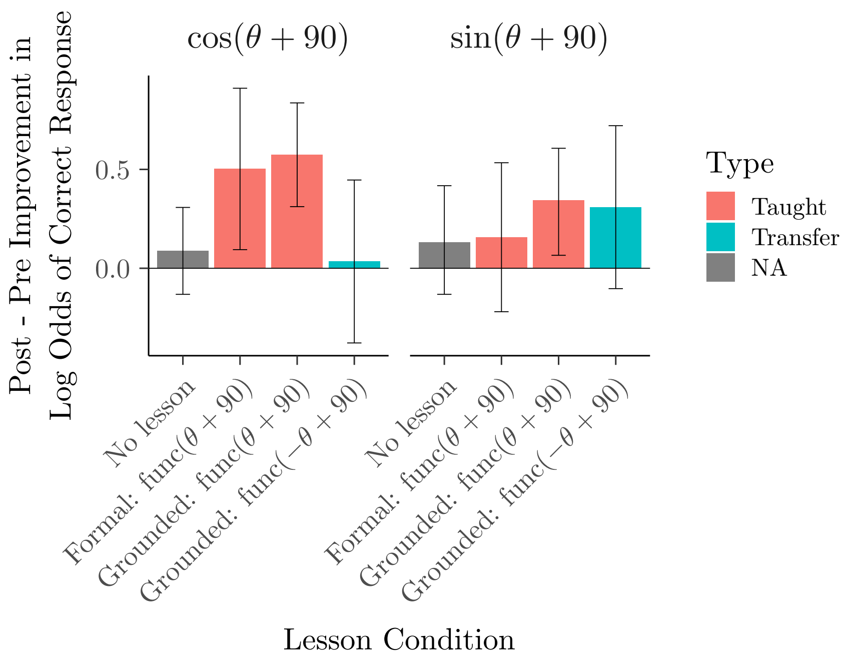


**Figure S6.** Mean improvement in log odds of responding correctly on $\text{func}(\theta + 90)$ problems from block 1 to block 2 (with 95% BCIs), split by function and lesson condition (no lesson from Study 1, formal lesson from Study 2, and grounded lesson from Studies 2 and 3). The grounded lesson condition was split based on the type of 90 problem presented in the lesson, with the grounded lesson of Study 2 grouped together with students in Study 3 who were taught $\text{func}(\theta + 90)$.

For this explanation to be sufficient, we would also need to consider behavior when the grounded lesson instead included $\text{func}(-\theta + 90)$ as taught problems. At first, we might question why there was such strong improvement as taught problems (from a starting point already high in accuracy), when students could have discarded or ignored their old strategies. During the lesson, however, in the course of learning to ground trigonometric expressions in the unit circle, students (assigned to this condition) saw the $\text{func}(-\theta + 90)$ problems, where their old strategies were relevant and potentially useful. Students could have integrated and grounded their old strategies in the unit circle. For instance, someone who used a right triangle to solve 90 degree problems may see the right triangle embedded in the unit circle representation, and these could provide mutual support in a virtuous cycle. They also received feedback, and so may have gained confidence in valid old strategies for these particular problems. These possible responses to the lesson are consistent with the improvement on taught $\text{func}(-\theta + 90)$ problems that students in this condition exhibited (shown in Figure S5).

The other problem type for which the grounded lesson seemingly failed to facilitate transfer was $\text{func}(\theta + 90)$. While we have previously been considering problem type by collapsing across function ($\cos$ and $\sin$), performance on this problem type seems to be sensitive to function. Figure!S6 shows improvement in accuracy by condition for this problem type, split by function. The grounded lesson appears to facilitate transfer on $\sin(\theta + 90)$ at least to the same approximate magnitude as performance when taught. On $\cos(\theta + 90)$, however, students show relatively large improvement when this problem is taught, and little to no improvement when this problem is held out for transfer.

One possible explanation for this disparity rests on the idea that the brief grounded lesson is not a wholesale replacement of strategies employed by students. Just as some students may have retained valid old strategies for $\text{func}(-\theta + 90)$, some students may have also retained invalid old strategies. One common invalid strategy that students applied in block 1 was the “pulling out the minus sign” heuristic, as discussed

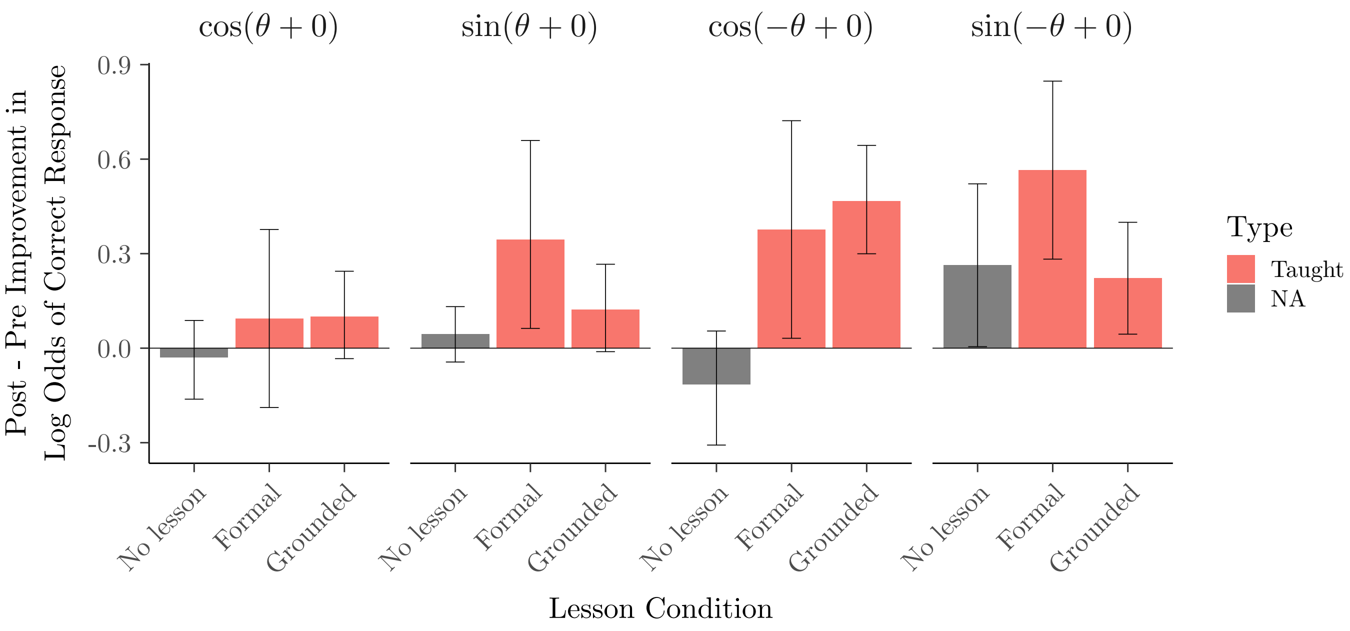


**Figure S7.** Mean improvement in log odds of responding correctly on 0 problems from block 1 to block 2 (with 95% BCIs), split by problem type and lesson condition (no lesson from Study 1, formal lesson from Study 2, and grounded lesson from Studies 2 and 3). The grounded lesson condition includes the students assigned to the grounded lesson in Study 2 and all students in Study 3.

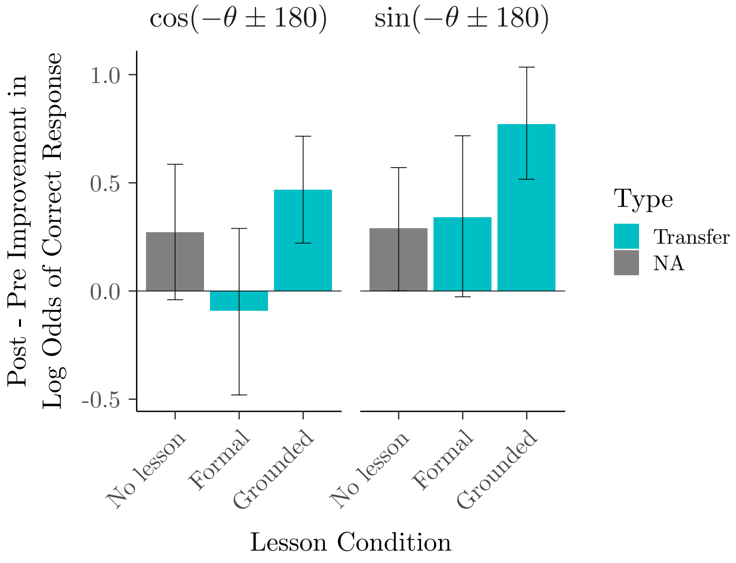


**Figure S8.** Mean improvement in log odds of responding correctly on func$(-\theta \pm 180)$ problems from block 1 to block 2 (with 95% BCIs), split by function and lesson condition (no lesson from Study 1, formal lesson from Study 2, and grounded lesson from Studies 2 and 3). The grounded lesson condition includes the students assigned to the grounded lesson in Study 2 and all students in Study 3.

previously. This heuristic was most apparent in the $\cos(-\theta + 0)$ problem, in which the correct answer was $\cos(\theta)$ but many students responded $-\cos(\theta)$. Figure S7 includes this problem, as well as other problems with a shift of 0, conveying the complete pattern of results in conjunction with Figures S4 and S5. While the grounded lesson does support learning to solve the $\cos(-\theta + 0)$ problem, there are two pieces of evidence which may temper our view of this success. First, the formal lesson causes nearly the same

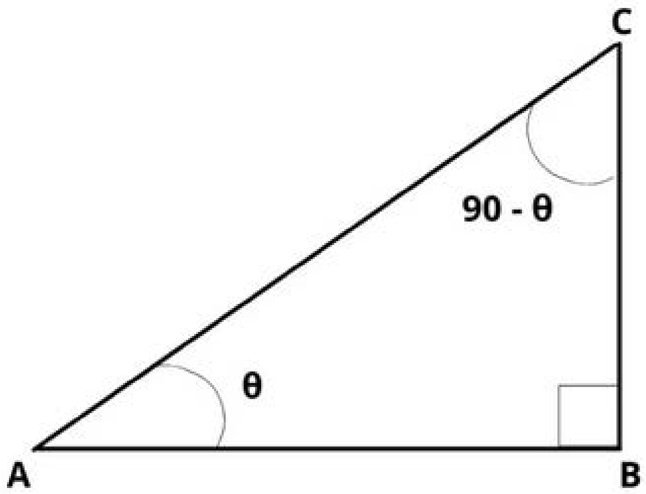


**Figure S9.** Diagram accompanying the explanation for the trigonometric identity $\sin(90 - \theta) = \cos(\theta)$. See text in Section 2.7.

magnitude of improvement in learning to solve this problem. Secondly, students in the grounded lesson continued to respond $-\cos(\theta)$ at an alarming frequency of 40%, 95% BCI [32, 47], $SD = 0.38$.

If students struggled with the sign of $\cos(-\theta + 0)$, those struggles could extend to other problems in which the sign of the argument to the trigonometric function (in the original probe expression or in an intermediate expression) conflicts with the sign of the correct answer. For instance, the problem $\cos(\theta + 90)$ involves a sign conflict because the correct answer $-\sin(\theta)$ involves a negative sign not present in the original expression.

Another problem type with a possible sign conflict is $\cos(-\theta \pm 180)$. The correct answer is $-\cos(\theta)$, but many students may be tempted to answer $\cos(\theta)$. They may be aware that $\pm 180$ results in changing the positive value to a negative value, and go on to reason (incorrectly) that the negative sign in front of $\theta$ reverses this result. Figure S8 shows performance on these problems as well as $\sin(-\theta \pm 180)$, broken down by lesson condition. While the grounded lesson does facilitate transfer for both cosine and sine, such facilitation appears to be weaker for cosine. (Note that this is also true in the formal lesson, albeit lower overall improvement in accuracy.) Or, in other words, the grounded lesson is facilitating transfer generally, although students are sensitive to characteristics of problems that may present difficulties.

### 2.7 A grounded explanation of the relationship $\sin(-\theta + 90) = \cos(\theta)$

Here we present a visuospatially grounded explanation of the trigonometric identity $\sin(-\theta + 90) = \cos(\theta)$. Consider the right triangle $ABC$ in Figure S9. The sum of the meaures of the two non-right angles in a right triangle must be 90 degrees, so if one of the angles equals $\theta$, the other always equals $(90 - \theta)$. The cosine of an angle is defined as the length of the leg of the right triangle adjacent to the angle divided by the length of the hypotenuse. Angle $A$ has measure $\theta$, the leg adjacent to $A$ is $AB$ and the hypotenuse is $AC$, so $\cos(\theta) = \frac{\overline{AB}}{\overline{AC}}$. The sine of an angle is defined as the length of the leg of the right triangle opposite the angle divided by the length of the hypotenuse. Angle $B$ has measure $90 - \theta$ and the leg opposite $B$ is leg $AB$, so $\sin(90 - \theta)$ also equals $\frac{\overline{AB}}{\overline{AC}}$. In other words, in any right triangle, $\sin(90 - \theta)$ and $\cos(\theta)$ correspond to the ratio of the length of the leg adjacent to the angle with measure $\theta$) to the length of the hypotenuse, and are therefore always equal. Similarly, $\cos(90 - \theta)$ and $\sin(\theta)$ always correspond to the ratio of the length of the leg opposite to the angle with measure $\theta$ to the length of the hypotenuse, and are therefore always equal as well.

Table S6. Parameters for a logistic mixed model using self-reported problem-specific use of unit circle and rule or formula and transfer (vs. taught) to predict whether each trial was answered correctly in block 3 in Study 3.

| Predictor | *b* | 95% CI | *p* |
|---|---|---|---|
| (Intercept) | 1.04*** | [0.56, 1.53] | $<$ .001 |
| Self-reported specific use of unit circle | 0.38 | [-0.17, 0.92] | .173 |
| Self-reported specific use of rule / formula | 0.80** | [0.32, 1.27] | .001 |
| Transfer (vs. taught) | -0.15 | [-0.41, 0.10] | .244 |
| Circle $\times$ Rule/Formula | -0.87** | [-1.47, -0.27] | .004 |
| Circle $\times$ Transfer | -0.11 | [-0.41, 0.20] | .489 |
| Rule/Formula $\times$ Transfer | -0.08 | [-0.39, 0.23] | .620 |
| Circle $\times$ Rule/Formula $\times$ Transfer | 0.25 | [-0.09, 0.60] | .148 |

## 2.8 Effects of rule and circle use on accuracy in Study 3

To examine the effects of rule and circle use on accuracy in Study 3, we used a logistic mixed model to predict whether each trial was answered correctly, based on self-reported problem-specific use of unit circle and rule or formula and transfer (vs. taught) in block 3, the block in which problem-specific self-reports were collected, in Study 3. The model and fitted parameters are shown in Table S6.

# 3 TRANSCRIPTS FOR $\cos(20 + 180)$ FROM THINK-ALOUD PROTOCOL STANFORD STUDY 3

## 3.1 Students Who Relied "Not at All" on Circle and "A Lot" on Rule/Formula

**Student #3 (Taught)**

STUDENT: And then here there's a rule in the lesson that said if you rotate it by 180 then it would be negative, so it would just be negative cosine of 20.

**Student #10 (Taught)**

STUDENT: This one here I think is that cosine 20 when you plus 180, same as minus cosine 20.

EXPERIMENTER: Okay, so do you have to like, visualize anything? You just know that at this point, but like, even right after [crosstalk 00:01:06] the lesson, you were just like, okay, I know that that's the relevant . . .

STUDENT: Yeah, I basically after the lesson, I just try to remember the rules. Didn't visualize anything at all.

EXPERIMENTER: Okay.

**Student #14 (Transfer)**

STUDENT: It's a 120 so it's going to be a cosine as well. It's going to reflect 20 over into the, that would be fourth quadrant, so now it's negative.

EXPERIMENTER: All right.

**Student #16 (Taught)**

STUDENT: Oh for this, for this one I actually visualized, so I usually imagine a arm, like a vector that just keeps rotating around. So for this one I imagine the vector is rotated to 180 and then I add another 20 degrees to it. And again, since cos in the third quadrant is negative I got the answer as minus cos 20.

EXPERIMENTER: Okay. And you used to figure out as negative, you're using "all silver tea cups?"

STUDENT: Yeah. So basically the way it works is A is all so everything in the first quadrant is always positive. S stands for sine so everything in the second quadrant is negative apart from sine. And then T is tangent so everything in the third quadrant apart from tangent is negative. And then the fourth quadrant is cups which is cos and everything in the fourth quadrant is negative apart from cos.

EXPERIMENTER: Yeah. And so when you visualize the 20 plus 180 and you're in the third quadrant, you then think to the mnemonic or-

STUDENT: Yeah.

EXPERIMENTER: Okay.

STUDENT: So I imagine where the angle is and then I think to the mnemonic. But also sometimes I just look at the answers and I'm like . . .

EXPERIMENTER: And something just feels right?

STUDENT: Yeah. What's that? Thinking fast you know like system one wins.

EXPERIMENTER: Yeah.

**Student #19 (Taught)**

STUDENT: Cos 180 plus 20 minus cos 20 equals cos 60 plus 180 is minus cos theta.

EXPERIMENTER: Okay.

**Student #22 (Taught)**

STUDENT: For this, basically other than the other ones that were odd or even, simple ones I basically use the same thing for all of them. So I just picture it on a unit circle and just say 180 plus 20 is still pretty close to the X axis so I know it's going to be a large negative number. So then I would just look, cross out the two that are positive. Look at the two that are negative and say, okay line D is close to the X axis, but quadrant one and say, okay cosine is going to be larger than sine at that point. And so I would pick that.

EXPERIMENTER: Okay. And you kept doing that logic or was there some point near then end where you were like, oh I know this one, or. . .

STUDENT: No, I just kept using that logic.

EXPERIMENTER: That's good.

**Student #25 (Transfer)**

STUDENT: Okay, so now, it's going to be 20 plus [180], so I know it's going to go basically shift right across that [rotates hand], a straight line across the axis, and then it said that when you have [180] plus 20, it's just pretty much negative of whatever the other angle is, so it's going to be negative cosine of 20.

EXPERIMENTER: Alright, and again, you can answer pretty quickly using that rule, but you know how to visualize?

STUDENT: Yeah.

**Student #36 (Taught)**

STUDENT: OK. Changing the 180 changes sign so this will be the same as negative cos 20.

EXPERIMENTER: And how do you know that the 180 changes the sign?

STUDENT: Because I use the graph visualization and on the graph it goes like that on both sides and we're dipping into the negative region. Cos of 20 is +cos of 20+180 would be negative because of the region it's in. So [inaudible 00:02:06]

EXPERIMENTER: OK. Not just as obviously it's negative and cosine of 20 is positive but also it's exactly halfway. And all this, these graphs, it sounds like you're quick enough that you know the answer without having to visualize like maybe you did at first but then it sounds like you know the answer, you didn't need to visualize anything at least at this point? Student: I could justify it to myself but it's funny, at the very beginning I didn't remember a lot of the rules and I didn't feel like thinking well because I usually like to draw the circle and figure it out but here I was just guessing. Then after there was some review on the rules I remembered why the rules worked the way they did so I just remembered and was able to answer.

EXPERIMENTER: You prefer the waves over - well you might start with the circle but then you liked the waves as intermediate maybe?

STUDENT: I'm very visual so I didn't like they didn't have paper. I didn't feel like [inaudible 00:03:11] So I did really try it this time. But after the review and getting to see the circles and thinking of the graph I remembered how the rules worked.

EXPERIMENTER: You didn't end up writing anything? It's OK if you did.

STUDENT: No it said not to use paper and pencil so I didn't write anything.

**Student #42 (Taught)**

STUDENT: Okay, I believe this was a formula too, we were given, and the C, and what we were shown. So this is cosine, this is minus cosine 20 I guess? Direct formula again

EXPERIMENTER: And yeah, you're just remembering the rule and applying?

STUDENT: Yeah.

**Student #54 (Transfer)**

STUDENT: Oh. For this one I'd do the same thing, I'd kind of just visualize a 20 degree angle and then add 180 to go this way which would be a negative cosine 20 angle.

EXPERIMENTER: And for both of those, do you feel as you went with more and more of those problems at some point, did you go, "Oh, I know the answer to this one." Or you're still visualizing [crosstalk 00:01:05]

STUDENT: I definitely, for the 180, I think were a lot easier for me because I could just kind of flip the negative positive for both.

**Student #57 (Taught)**

Student: I go over to 180, so starting from zero to 180, then I know I add 20, and in that case it's the same as cosine 20, but because it's in the third quadrant, it's negative, so it's negative cosine 20.

Experimenter: Alright, and for both of those problems, or either of them, or ones like them, you're visualizing on the unit circle. Did you get to some point where you're like, "Oh, I know the answer to this one," or was it you're doing that procedure through the whole- Student: It's usually through the whole but then for some if I know if it's 90 plus something, I know it's definitely cosine. It just goes straight to the quadrant and I know with sine it stays cosine, but positive if it's cosine, it stays sign and it changes directions so, there are things because I've been doing them for so long, I can just go straight ahead.

Experimenter: I gotcha.

**Student #58 (Transfer)**

STUDENT: cos(20 + 180)… so it's negative cosine 20 and I choose that because… I just know that plus or minus 180 flips the [hand over hand motion] sign [fingers switching motion]. So theta, if you have cosine or sine theta plus or minus 180, then it's gonna be negative cosine or sine.

EXPERIMENTER: And that's again …

STUDENT: [crosstalk] another rule …

EXPERIMENTER: [crosstalk] the rule from the last time, or maybe even before, and you're not really visualizing anything to do that?

STUDENT: [crosstalk] No, it's just like …

EXPERIMENTER: [crosstalk] You just know to flip the sign.

STUDENT: Yeah.

**Student #62 (Taught)**

STUDENT: Yeah, and then, this one so I think about the angel and you go cosine 20 N plus 180 and then I don't know. Cause on the sine you have cosine, you'd have sine, whatever and if you go 180, it's just a negative sine. But for cosine, I wasn't sure if it switched to sine or if it stayed cosine and went to negative.

EXPERIMENTER: How did you know that - you were saying that if it was sine of 20+180 you would know the answer?

STUDENT: Yeah.

EXPERIMENTER: And if it was a negative sine of 20 and how do you know that? Is it like a rule or [crosstalk 00:02:46] is it something, you were talking about visualizing the-[crosstalk 00:02:51]

STUDENT: Visualizing it , it's like the opposite point so like you switch it to negative.

EXPERIMENTER: Okay.

STUDENT: But on this one,

EXPERIMENTER: And so the sine is just like the point on the circle or does it have some other meaning in terms of ones I'm visualizing on the angle of point plus 180?

STUDENT: I just think of it as a point on the unit circle [crosstalk 00:03:11]and then just see the opposite of it. [crosstalk 00:03:12] But this one -

EXPERIMENTER: The cosine has a little trick, [crosstalk 00:03:19], you're not sure what, [crosstalk 00:03:21] where cosine it [crosstalk 00:03:20]corresponds to-

STUDENT: What it does, so I mean I probably just guess negative. I don't know cause I don't know, I was thinking about the rule, I thought it changed it to sine in the opposite, so I would just do that. [inaudible 00:03:36]

EXPERIMENTER: You feel like for this type of problem, after the lesson, do you feel like you would just systematically choose negative sine or a particular answer or[crosstalk 00:03:45] is it like, well, this one I'm really not sure so I'll guess between some of them?

STUDENT: I feel like if looked at the lesson again, I would know. But I don't remember so I would just systematically guess the opposite and negative.

EXPERIMENTER: Okay. You may have to check [inaudible 00:04:15]

STUDENT: No, um

EXPERIMENTER: Hit next.

## 3.2 Students Who Relied "A Little" on Circle and "A Lot" on Rule/Formula

**Student #5 (Transfer)**

STUDENT: Since you're adding 180, you basically take the opposite of what the original angle is, so it will be the negative cosine of 20, because when you flip it across the circle, you change sign, but everything's the same function.

EXPERIMENTER: And this one you know fast enough by now, like are you still visualizing it at the end or is it that you're like okay, I know this one by the end?

STUDENT: I think I still visualize it. I know it's going to be in the negative X and negative Y direction, but I don't really focus as much on where that angle actually is.

EXPERIMENTER: That makes sense.

STUDENT: So negative cosine 20 …

**Student #7 (Transfer)**

STUDENT: Didn't really think of the unit circle because I don't think I need to do that yet but for these kind of problems, then I would have to think of it a little. Still, I'm more focused on the formula.

EXPERIMENTER: Was that … At the beginning, were you thinking more one way, and now you're thinking more another, or through the whole thing?

STUDENT: For the previous question?

EXPERIMENTER: Yeah. Well, I guess … This one, you're saying you might visualize a little but now, you're also still …

STUDENT: Yeah.

EXPERIMENTER: For a 180 problem, you know the rule now, but I was thinking was that something that maybe changed over time, with practice, or that you knew [crosstalk 00:01:03].

STUDENT: Yeah. Initially, I was more still explicit rules and formula, but, after a while, then, I feel like it might help better to visualize the circle, so yeah. Pretty much. It's been a while since I've done trig. It's so …

EXPERIMENTER: No, it's okay.

STUDENT: It's terrible, the first one I did. Yeah, for this one, I would think of it as a circle and then, at first, it's the 20 degree angle, and then switch to 180 over here.

EXPERIMENTER: Right.

STUDENT: Yeah. Then …

EXPERIMENTER: What was the initial rule that you were going off of or …

STUDENT: The explicit rule?

EXPERIMENTER: Or maybe you didn't have one that . . .

STUDENT: It was just anything with a cosine and 180, then, is just the negative . . . Hang on. Let me think. Yeah, so it's the other way around, right? Oh, dear.

EXPERIMENTER: Oh.

STUDENT: Yeah. Even now, I'm still a little bit fuzzy because that was a lot of things that . . . Well, yeah, I guess going by the explicit formula doesn't help as much as when I'm thinking of it as a circle. Yeah.

EXPERIMENTER: All right.

STUDENT: Cosine 120 . . . No, 180, so . . . Hang on. That's cosine . . . Negative cosine 20?

EXPERIMENTER: Okay.

STUDENT: I'm sorry. I'm terrible at these.

EXPERIMENTER: No. No, no need for apology. Just tell me what you're thinking.

**Student #18 (Transfer)**

STUDENT: And then for this one I'm also imagining a unit circle. First I go to 28 degrees and then I rotate it by 180. And then in this case it's equal to the negative cosine 20 because the horizontal component is being flipped.

EXPERIMENTER: All right.

**Student #23 (Transfer)**

STUDENT: Cosine of 20 plus 180 is the same as negative cosine 20 according to the rule that was taught in between the two lessons. I mean, I could go through the unit circle as well, but that's quicker.

**Student #28 (Taught)**

EXPERIMENTER: All right.

STUDENT: Cosine 20 plus 180, I imagine seeking the angle 20 degrees and then 180 and that flips it so it's negative cosine 20.

EXPERIMENTER: And for both of those problems you're even at the end you were still doing the . . . visualizing the same kind of procedure or any of them you felt like, "Oh, I know this answer, I don't need to do this again." You know?

STUDENT: Some of them. Like when I get in the flow, I begin to see, like, oh, it's like plus 180, I just flip the sign.

EXPERIMENTER: Yeah.

STUDENT: I guess like I was going slower because I was trying to explain my way through it. I guess sometimes with the zero ones I just do the same angle except for I think sine sometimes because the negative sign strays over. So sometimes I get caught up on that.

**Student #34 (Taught)**

STUDENT: 180. Okay. Lemme actually try hard for this one. So, it's like 20. So, on the circle, that's in the positive area. So, that's above the horizontal axis and then you go 180 from that so that would lead you to a negative 20. So that'd be cosine. Negative 20.

**Student #41 (Transfer)**

STUDENT: Here we have 20 degree plus 180, so the 180 brings it to the totally opposite quadrant. Which will cause that 20 degree angle to result in a negative output from the cosine function, so that'll just be negative 20.

**Student #49 (Transfer)**

STUDENT: So, cosine one-eighty you're going all the way around and then you go an extra twenty, bump that's gonna be on the left side of the graph, so that's negative, that's also going to be a cosine rather than a sine.

EXPERIMENTER: For these last two, you're describing using a unit circle, do you feel like, at least after the lesson that you're pretty confident and so you didn't have to visualize every part, you're like I know the answer to this one?

STUDENT: Kinda, I'm also using a little bit of just thinking I know phase shift, but I'm not actually writing it out so I'm moving things around in my head a little too fast, I think. Cause ... yeah. If that makes any sense.

EXPERIMENTER: So like the cosine of negative fifty part you were thinking was negative cosine[crosstalk] and actually think its positive on the unit circle.

STUDENT: Yeah, yeah, cause I was doing sine and I was like its going down, but its actually positive, so... moving too fast for my own good.

**Student #51 (Taught)**

STUDENT: So cosine 20 plus 180. I know that when it's 180 the cosine stays the same. Twenty is positive so I'm gonna assume the answer is cosine twenty. Might have been wrong.

EXPERIMENTER: That was a rule, given if there's a 180? Is that right?

STUDENT: Yeah, that's the way I usually remember it. Now that I'm thinking about it, it might have been negative, because it changes direction I think. I just remember that when it's 180 the identity of the sign will stay the same so cosine or sine it'll stay the same, and then when it's 90 it'll change between sine and cosine. Yeah I mess up about whether it's positive or negative though, 'cause I can't remember that rule.

EXPERIMENTER: Do you feel like for that type of problem where, I think it was cosine of 20 plus 180 or something, you're a little unsure right now but at least before you were consistently saying positive cosign of 20, is that right?

STUDENT: Mm-hmm (affirmative)

**Student #59 (Taught)**

STUDENT: Here we have cosine of 180 plus 20. First, I'm going to go 180 degrees. And then I'm going to go another 20 degrees, it would put us in the third quadrant. Again, by the pneumatic "all students take calculus", cosine and sine are both negative in that quadrant. But we only care about the cosine, so I would say that this is a negative cosine of 20.

EXPERIMENTER: Alright. For both those problems, that strategy was basically what you're doing throughout or was it like after seeing a number or the problems, I know the answer to this one without having to visualize the angle, for instance.

STUDENT: Ahh. No I would always go through those steps. I wouldn't remember... answers from previous...

EXPERIMENTER: Okay.

**Student #61 (Transfer)**

STUDENT: Yeah. Okay, so cosine 20, so first imagine cosine 20, the angle, and then plus 180, so it's going to be 180 degrees from there. Then that would be equivalent to I believe negative cosine of 20.

EXPERIMENTER: Why is that?

STUDENT: Because it's pretty much the same angle as cosine of 20, it's just in a different quadrant, so you flip the sine.

EXPERIMENTER: Okay, and where is the cosine? Or what's the cosine mean in this one? You have the cosine of 20 plus 180.

STUDENT: So in this case, it would be ... adjacent over hypotenuse, and then plus 120 is going to be ... it's going to be the same parallel line, but you're going to be pretty much coming from ... because it's equivalent to cosine of 200. What was your question again?

EXPERIMENTER: No, that's all right. That helps, I think.
STUDENT: Yeah.

### 3.3 Students Who Relied "A Lot" on Circle and "A Lot" on Rule/Formula

**Student #1 (Taught)**

STUDENT: Cos 20 plus 180, we go by 180 and then go down by 20. It's the same except it's on the negative side, so it's minus cos 20.

**Student #4 (Taught)**

STUDENT: For this one, because there's plus 180, I would picture the other degree first, which is what they taught me in the study, so I picture 20 degrees in the first quadrant. And then picture that rotating 180 degrees, which is in the third quadrant, which would be negative cosine value.
STUDENT: So that would be negative cosine of 20.
EXPERIMENTER: Mm-hmm (affirmative).

**Student #12 (Taught)**

STUDENT: So for this I just know that when you add 180 you're just gonna get the opposite result, and so I know cosine 120 is positive, so I would get the negative back.

**Student #15 (Taught)**

STUDENT: Yeah. This one with going of the 180. First, you kind draw this out, like start from zero and sweep across 180. Add 20 more degrees so they're ... you would be at this cosine, like a 20 degree angle right here. In the third quadrant, so that cosines gonna be negative, but it will still be the same as if you were looking at a cosine of 20 degrees right here. So the cosine would be the same. But it would be negative because it's on the fourth quadrant, or the third quadrant.
STUDENT: So then negative cosine 20.
EXPERIMENT: Alright. Keep going.

**Student #29 (Taught)**

STUDENT: Okay so, when there's 180 it just kind of... flipity flops it, no matter if it's sine or cosine. So cosine of 20 plus 180 is the same as negative cosine of 20.
EXPERIMENTER: Okay, so for that one you are using the "flipity flopity" rule.
STUDENT: Yeah.
EXPERIMENTER: So you don't need to really visualize anything for that one.
STUDENT: Um, I guess I still kind of think of rules and steps on some of them, but I still always pop it up with that unit circle, so here (horizontal hand on right) and then flipity flopity it (moves hand to left).
EXPERIMENTER: So you know that it's on the other side of the circle.
STUDENT: Yeah.
EXPERIMENTER: And so that helps for you to remember maybe partly the flipity flopity part.
STUDENT: Yeah, exactly.

**Student #30 (Taught)**

STUDENT: And then, for this one, I go over 180 on a unit circle, and I know that's basically another 90 degree angle. And so if I'm adding 20, I know I need to do a sine 20, but in the third quadrant, cosine here is negative and sine is negative. So I don't really need to do negative sine 20; I can just do ... no. I need to do negative sine 20. Yeah. Because they're both negative. Okay.

EXPERIMENTER: I'm not sure if I totally follow you here. So if I follow you, you found out the angle of 20 plus 180. And then you're trying to map it onto the answers, and I didn't really follow you about how you got to the answer there.

STUDENT: Oh, okay. So one of the formulas was that if you have ... the sine of 90 minus an angle is equal to the cosine of that angle. So I know 180's just another 90-degree angle. And so if I just make that 90 and I'm adding 20 to it, I know I can do sine of 20. But because cosine in the third quadrant of a unit circle is negative, I did negative sine 20.

EXPERIMENTER: Okay.

**Student #37 (Transfer)**

STUDENT: So this is ... You're going to swing all the way around, and you're going to continue down. So, now this is before the x and y, which means that it's now a negative cosine, but it's still a cosine of 20. So, it's negative cosine of 20.

EXPERIMENTER: All right.

**Student #45 (Taught)**

STUDENT: I know that one eighty brings it back to, it's the same idea as the zeroes it still brings it back to twenty.

EXPERIMENTER: Say that again.

STUDENT: Cosine of one eighty would not impact adding it on into the angles, it would be the same, it would be cosine twenty again, so cosine twenty plus one eighty would just be cosine twenty.

EXPERIMENTER: Okay, and that's just another rule from the lesson?

STUDENT: From the lesson, yeah.

EXPERIMENTER: Let's try again, or check it.

**Student #63 (Taught)**

STUDENT: I think. I've been using a rule of thumb picturing a circle where if you add a hundred and eighty degrees it's just like, flipping the angle across the circle. So negative cosine twenty.

EXPERIMENTER: And when you flip it around in the circle, like I get how you flip the angle, how do you get the cosine from that, or are you thinking "Well, since the angle is flipped, then I have to flip the cosine?". Is that right, or?

STUDENT: Oh, I guess, do you mean, why is the positive negative? I guess I assumed that if you add a hundred and eighty degrees to an angle the only thing that changes is the positive or negative sign. I don't know if that was explicitly given.

### 3.3.1 Students Who Relied "Not at All" on Circle and "A Little" on Rule/Formula

**Student #32 (Transfer)**

STUDENT: So 180 is on the unit circle a complete flip of what it is and so that's gonna make it a negative cosine. Something to go with negative cosine 20.

EXPERIMENTER: Why is that?

STUDENT: Because if you think about it flip [inaudible] circle and then the cosine is the measurement of the axis then that is now negative.

**Student #38 (Taught)**

STUDENT: Correctly. Okay, so that's cosine of 20 plus 180, so I think the rule is when you add 180, it becomes negative, so I think that's cos of negative 20, which is [inaudible 00:00:50] I think it's negative cos of 20, yeah.

**Student #44 (Transfer)**

STUDENT: I remember that it was like, if it's plus 180, then it's like posing. So sine of the, the negated sine of the same angle.

EXPERIMENTER: Okay. And hold on, so the first one was cosine of negative 50 plus 0.

STUDENT: Yeah.

EXPERIMENTER: And you said cosine of 50.

STUDENT: 50, yes.

EXPERIMENTER: And if it was sine of negative 50 plus 0, it's the same rule or is there a different ... ?

STUDENT: I think it's still just sine.

EXPERIMENTER: Okay.

STUDENT: Yeah, so same rule applies, I think.

EXPERIMENTER: Okay.

STUDENT: But I think it's not negative. Or, it is negative sine if it's like ... So it's like 50 plus 0 so negative sine 50, I think was the rule. I'm not really sure.

**Student #50 (Transfer)**

STUDENT: Cosine 20 plus 180. So, 180 ... So if I go ... I'm imagining a unit circle, and if I rotate around 180 degrees, then I assume I get the negative, so that's why I'm putting negative cosine 20.

EXPERIMENTER: Okay, and ... hold on. So ... That one you were visualizing a unit circle ...

STUDENT: Yeah.

EXPERIMENTER: And the previous one, it was pretty simple, so you don't bother visualizing? Is that that? Or you did at first, but then you're like ...

STUDENT: I didn't bother visualizing.

**Student #52 (Transfer)**

STUDENT: This one you're gonna go the other way and it's gonna change the, so in the same circle, it's gonna go the other way. It's gonna be negative.

### 3.4 Students Who Relied "A Little" on Circle and "A Little" on Rule/Formula

**Student #27 (Taught)**

STUDENT: This one, I picture having the angle and then flipping it across the unit circle, basically, so that would be a negative and switched, so it would be negative cosine 20. Is that enough of an explanation?

EXPERIMENTER: Sure.

STUDENT: Okay.

EXPERIMENTER: Yeah.

STUDENT: This one-

EXPERIMENTER: What was the answer to that one?

STUDENT: Negative cosine 20.

EXPERIMENTER: Okay.

STUDENT: Or negative sine 20. Whatever the original sign was, it's the opposite.

**Student #39 (Transfer)**

STUDENT: So here I see as 180 going all the way over to the first quadrant, and then continuing on 20 more degrees which would be negative for a cosine, so negative cosine 20.

**Student #46 (Taught)**

STUDENT: And then, I guess right now I'm picturing the unit circle, like cosine, like an angle 20, and then 180 would just like move it to the other side. So I don't think I would do anything. It would still be cosine 20.

EXPERIMENTER: Say that again.

STUDENT: So I think that ... So with the angle 20 and then plus 180 would just move it to the other side which would just like be the same. I can't remember if cosine is down or if it's this way, so I'm just gonna go with ... It's either negative cosine 20 or cosine 20. But because the negative inside the circle doesn't matter, or inside the parentheses doesn't matter, I'm just gonna guess that it's gonna be this way.

**Student #55 (Taught)**

STUDENT: This one, before I used to do it cosine to 100 and then did the, what's that called? The graph and how they're certain parts of the graph where it's positive or negative. So, like, since this is cosine 200, it would be in the tangent area. So, it's going to be negative, so I deduced it to negative sign 20 and negative cosine 20.

EXPERIMENTER: Yeah.

STUDENT: And then this is usually where I get stuck, so I have a 50/50 chance and then I remember, like, cosine 20 is, like, a long thing. It's all on the x axis, so it's going to be long. A larger number than sign 20. So, then I use that to figure out the relative value for cosine 20 and I usually get negative cosine 20.

EXPERIMENTER: So, I get that you're trying to think, okay, cosine is 20. Has a bigger magnitude than a sign of 20, but I'm confused about how do you figure out for the angle ... The 200 degree angle? How does knowing one's ... the cosine big sign of 20 ... Do you compare that to 200 or something?

STUDENT: In my math class before, we had this ... We would do the graph and then it would say, like, all students take calculus.

EXPERIMENTER: Yeah.

STUDENT: Since the 200 degree is in the tangent area, so it's going to be a negative value. So, I knew that I had to be negative sign 20 or negative cosine 20. And then I just thought relative, if cosine 20 was a lot like the other sign. The negative x axis part and then it will be the mirror image of it.

EXPERIMENTER: For a- Sorry. You were just talking about the 20 degrees there or are you comparing it to 200 degrees?

STUDENT: Yeah.

EXPERIMENTER: At the very end. That's what I'm trying to figure out.

STUDENT: Relative to the- Yeah. Comparing to 200.

EXPERIMENTER: Okay. And so, you were saying that the cosine of 20 is a big magnitude and then you said something was the mirror image?

STUDENT: Yeah, because since cosine of 20 is the same cosine negative 20, so it's going to be on the same thing. I just thought you flip it over to the next ... across the y axis.

EXPERIMENTER: Cool. And so, that one you, sort of, use a mix that all students take calculus and the units circle.

STUDENT: Yeah.

EXPERIMENTER: On the previous one, since you just know that rule, you don't need to do that?

STUDENT: Yeah.

EXPERIMENTER: But you could use the same strategy or not?

STUDENT: Yeah.

EXPERIMENTER: Okay.

STUDENT: You could use the same strategy. It's just, like, since zero-

EXPERIMENTER: It's easy.

STUDENT: Yeah.

**Student #60 (Transfer)**

STUDENT: So in this case, we're showing it's cos, it's 180, so it'd be all the way around plus 20 would be up here. So it'd be negative sin, or, sorry positive sin 20 because I'm doing it off of this.

EXPERIMENTER: And for that one, where did you start and how far did 180 take you?

STUDENT: Started here and 180 brought me all the way down here to cos, negative cos. It's 20, so you keep going 20 more. So it'd be negative cos 20.

EXPERIMENTER: Why is it negative?

STUDENT: Because we're on the bottom half of the circle.

### 3.5 Students Who Relied "A Lot" on Circle and "A Little" on Rule/Formula

**Student #6 (Transfer)**

STUDENT: And then, the cosine of 20 plus 180, so, flip to 180, and then go 20 and it should be same as cosine of 20.

EXPERIMENTER: Why was that?

STUDENT: Because it's ... so 180 and then 20 is the same as just 20, up there, yeah.

EXPERIMENTER: You're comparing the angles or something off the angles?

STUDENT: The angles. Yeah.

EXPERIMENTER: Okay.

**Student #13 (Transfer)**

STUDENT: This is just this one because there's like a formula thing where it's like if you add 180 it's like the same thing, but like negative, opposite version.

EXPERIMENTER: Okay. So that one you didn't have to visualize anything for?

STUDENT: Yeah.

EXPERIMENTER: And what about the first one? You were describing the circle [crosstalk] ...

STUDENT: Yeah I couldn't ...

EXPERIMENTER: And so you're still using that or is like more like you knew it but you're just like justifying?

STUDENT: That one, like I was describing how I normally do them but then I realized that I didn't need to do that.

EXPERIMENTER: Yeah.

**Student #31 (Transfer)**

STUDENT: Here we have cosine of 20 which takes us up. Then we add 180 degrees, so now we are on the negative side of the x axis which is negative cosine of 20.

**Student #47 (Transfer)**

STUDENT: So, cosine, its 20 degrees and you flip it to 180 and its gonna be a sine- so cosine but its gonna be negative.

**Student #53 (Taught)**

STUDENT: Yeah. Okay, this one, cosine 20 is like, there. Add 180 to it, it would shift over here. So then it would be the same as the cosine of that, would be the same as the negative cosine of 20, I believe. But honestly, I have no idea. I don't like trigonometry.

EXPERIMENTER: That's alright. So that one you were thinking maybe in terms of the unit circle. But, as you went, do you feel like you just knew that answer, or did that one - you would be visualizing?

STUDENT: Yeah, no. I didn't know it at all. I had to visualize it and I'm still not confident about it in any way.

EXPERIMENTER: That's alright. The previous one was like a cosine negative 50 plus 0. That one you had a rule where you did know that answer.

STUDENT: Yeah, just adding 0 to something.

EXPERIMENTER: Well, it's also the cosine of negative 50 being the negative cosine of 50? And that was a rule that was in the lesson or that you knew before, or both?

STUDENT: I already knew that. I think it was also in the lesson.

## 3.6 Students Who Relied "Not at All" on Circle and "Not at All" on Rule/Formula

**Student #8 (Transfer)**

STUDENT: So for this one, cosine 20 plus 180, I would envision cosine at 180, which goes from 1 to 0 to -1 [index finger moving along wave], and I add 20, so it goes up a little bit, so it's a little bit more than -1 so -0.9 or whatever it is. And then cosine of 20 is 0.9 but positive so I'd go with negative cosine 20.

**Student #26 (Transfer)**

STUDENT: So 20 plus 180, so I'd do sine minus 20.

EXPERIMENTER: Why is that?

STUDENT: I don't know. It feels right.

EXPERIMENTER: It's okay. You were doing some hand motions. You're trying to think of something?

STUDENT: Yeah, like a swivel. Like here, then it goes 180 degrees, which is to the other side of the circle.

EXPERIMENTER: Yeah, so you get to that spot on a circle and then you're not sure quite how that maps on to the answers but-

STUDENT: No. I just try and visualize the circles somehow. It always goes clockwise. I don't know.

EXPERIMENTER: No, that's alright.

**Student #40 (Transfer)**

STUDENT: Same thing. I know that this is cosine, so now I'm not exactly sure where cosine started, because I went through a bunch of the lessons before I remember that sine is one side and cosine is the other side. I know when you add 180 degrees, then it switches to the other side, which means it's a negative angle. Maybe it's negative from the X-axis. Would that be a negative sine 20? I don't really know. Maybe it could be. I guess if it's on that side, maybe it's a positive angle, but a negative … Oh wait, maybe that is a negative sine, because the sine's on the X and that's a positive sine and that's a negative sine. I could be totally wrong about this.

EXPERIMENTER: You're alright.

STUDENT: I am right?

EXPERIMENTER: No. Sorry I say that a lot, I guess like. It's okay, no need to apologize.

STUDENT: I'll do negative sine 20, because why not?

**Student #43 (Transfer)**

STUDENT: Cosine 180 it's a negative half of the x axis, go down twenty. Cosine's negative so it's negative cosine twenty.

EXPERIMENTER: Alright. And for both of those problems, as you went and saw more problems did you feel like, "Oh, I know the answer, I don't have to visualize this"? You might get it a little faster, but you're still visualizing?

STUDENT: For more complex ones, so if there was like 180 or something like that, I just kind of went through that and it was easy. But if it were like 80 plus 70 or I think I saw something like that, 80 + 70, then I would probably visualize that with an actual angle.

EXPERIMENTER: Okay.

**Student #48 (Taught)**

STUDENT: And then here it's all positive, so I'm just going to go cosine 20.

EXPERIMENTER: And both of these are tricks or rules or heuristics...

STUDENT: Yeah both are just little tricks, things that I noticed from reading through the in between where it was like [inaudible 00:01:12] unit circle. You'll see in my answers I didn't use unit circle or anything like that it was all just finding little tricks or I guess what I thought was a trick.

EXPERIMENTER: Were there some that you had trouble with, some that fit your rules or tricks but some types of problems that didn't or you felt like you had a response for every kind of problem?

STUDENT: Yeah I mean I felt like I had a response whether or not that they were right. That's a great question because I mean obviously the first round I didn't remember doing this at all because I did this in like middle school or something like that.

**Student #64 (Taught)**

STUDENT: Okay. So this one, I see that it's adding 180 so then I'll probably think it'll be the opposite but still cosine so this one.

EXPERIMENTER: Okay. And that's just, you're just

STUDENT: Because I remember in the diagram it like did the whole rotation and it was like on the opposite side.

EXPERIMENTER: Okay.

STUDENT: So negative one next to the one. Or something, I don't know, cause it's all one, right?

EXPERIMENTER: Okay. Yeah.

STUDENT: Alright, so, I'll put this.

### 3.7 Students Who Relied “A Little” on Circle and “Not at All” on Rule/Formula

**Student #17 (Taught)**

STUDENT: For here, you have to do the plus 20, and then plus half a circle. I guess it's minus sine 20, I'm not sure. I don't know what the results is. I can visualize it, but I don't know what the result is, because I'm still a bit unclear.

EXPERIMENTER: That's all right.

### 3.8 Students Who Relied “A Lot” on Circle and “Not at All” on Rule/Formula

**Student #2 (Taught)**

STUDENT: This one, so that's the same as, again, I'm using the unit circle thing. That's the same as negative cosine of 20, because you can swing to the negative X axis. Oh wait, I'm doing that opposite. It's still the same answer. Yeah, you swing to the negative of X, then you go 20 more. That projects the same, and just negative, so that's negative cosine 20.

EXPERIMENTER: All right.

**Student #11 (Transfer)**

STUDENT: So like that. And then, for this kind of problem, so cosine 20 plus 180, I would just swing my hand like 180 degrees, and then say “Okay, 20 more.” So the angle to the horizon, or to the horizontal is 20, so I

know that my answer will have a 20 in it, and it will be like cosine. So, when cosine's in this direction it's negative, so it's gonna be negative cosine 20.

**Student #21 (Transfer)**

STUDENT: And then so cosine 20 plus 180, so I would do the same thing: start at 0. Then I would go to 20 and then I would go all around to 180. So I would like do this in the room too, so I would start here, go to 20, and so 180 would flip it this way. So then I know I'm just evaluating this length here as well. So I know that this length here would be the same as cosine of if I were to kind of flip the circle, like cosine of 20 it would be the same length. But since it's on the other side, it would be a negative value because this is a positive side, this is a negative side, so I'd pick cosine of … negative cosine 20.

**Student #24 (Transfer)**

STUDENT: Cosine of 20 plus 180. I know 180 is just 180 degrees and then add another 20. I know that it could be negative since it's on that side of the x axis. And 180 plus 20, it would be just cosine of negative 20 I think, or negative cosine of 20.

EXPERIMENTER: Okay.

**Student #33 (Taught)**

STUDENT: Okay.

STUDENT: This one, I think I tried to think of the rule, it's just start at twenty, and go on eighty, it's just right on the other side of the unit circle. And now you are looking at a change from…

STUDENT: Again, yeah, so it goes into the negative area of the y-axis, but- Oh, shoot. It should just be negative cosine twenty, but I don't think that's right. I think it might be negative sine twenty.

EXPERIMENTER: Alright. You were saying, "Oh, this one, I know the rule for this," at first, but then you were sort of like, thinking a little slower with the [crosstalk 00:02:42] circle?

STUDENT: Well, just because I know the 180 just goes- I'm trying to flip it over, but then when I started thinking about it more, I'm like, if I stopped there, I would think just a negative cosine of twenty, so I feel like it's the answer, but then if I try and think about it more…

STUDENT: Yeah, no, that makes sense, because you start at cosine of twenty… Right there. Yeah, that number, and then that's gonna be the same angle if you just add 180 to it. It's gonna be negative. It's on the negative side of the unit circle.

EXPERIMENTER: Alright.

EXPERIMENTER: Go ahead.

**Student #35 (Transfer)**

STUDENT: So, now I draw a 20 degree angle, at 180 so it flips to the third quadrant of the unit circle, and basically when you go to the third, everything is negative, so I know that the answer has to have a negative sign in it. And also because this is the 180 flip, cosine stays the same as cosine, so I choose negative cosine 20

**Student #56 (Transfer)**

STUDENT: Yeah, so for this one I'm imagining it's 180. So 20+180, it would essentially be a negative answer. So it's like 180 and then 20. And then it would be, you would want this answer so it would be negative cosine 20, I think.

EXPERIMENTER: Yeah.
